# Regional conformational flexibility couples substrate specificity and scissile phosphate diester selectivity in human flap endonuclease 1

**DOI:** 10.1093/nar/gky293

**Published:** 2018-04-30

**Authors:** Ian A Bennet, L David Finger, Nicola J Baxter, Benjamin Ambrose, Andrea M Hounslow, Mark J Thompson, Jack C Exell, Nur Nazihah B Md Shahari, Timothy D Craggs, Jonathan P Waltho, Jane A Grasby

**Affiliations:** 1 Centre for Chemical Biology, Department of Chemistry, Krebs Institute for Biomolecular Research, The University of Sheffield, Sheffield S3 7HF, UK; 2 Department of Molecular Biology and Biotechnology, Krebs Institute for Biomolecular Research, The University of Sheffield, Sheffield S10 2TN, UK; 3 Manchester Institute of Biotechnology, School of Chemistry, The University of Manchester, Manchester M1 7DN, UK

## Abstract

Human flap endonuclease-1 (hFEN1) catalyzes the divalent metal ion-dependent removal of single-stranded DNA protrusions known as flaps during DNA replication and repair. Substrate selectivity involves passage of the 5′-terminus/flap through the arch and recognition of a single nucleotide 3′-flap by the α2–α3 loop. Using NMR spectroscopy, we show that the solution conformation of free and DNA-bound hFEN1 are consistent with crystal structures; however, parts of the arch region and α2–α3 loop are disordered without substrate. Disorder within the arch explains how 5′-flaps can pass under it. NMR and single-molecule FRET data show a shift in the conformational ensemble in the arch and loop region upon addition of DNA. Furthermore, the addition of divalent metal ions to the active site of the hFEN1–DNA substrate complex demonstrates that active site changes are propagated via DNA-mediated allostery to regions key to substrate differentiation. The hFEN1–DNA complex also shows evidence of millisecond timescale motions in the arch region that may be required for DNA to enter the active site. Thus, hFEN1 regional conformational flexibility spanning a range of dynamic timescales is crucial to reach the catalytically relevant ensemble.

## INTRODUCTION

**Figure 1.**
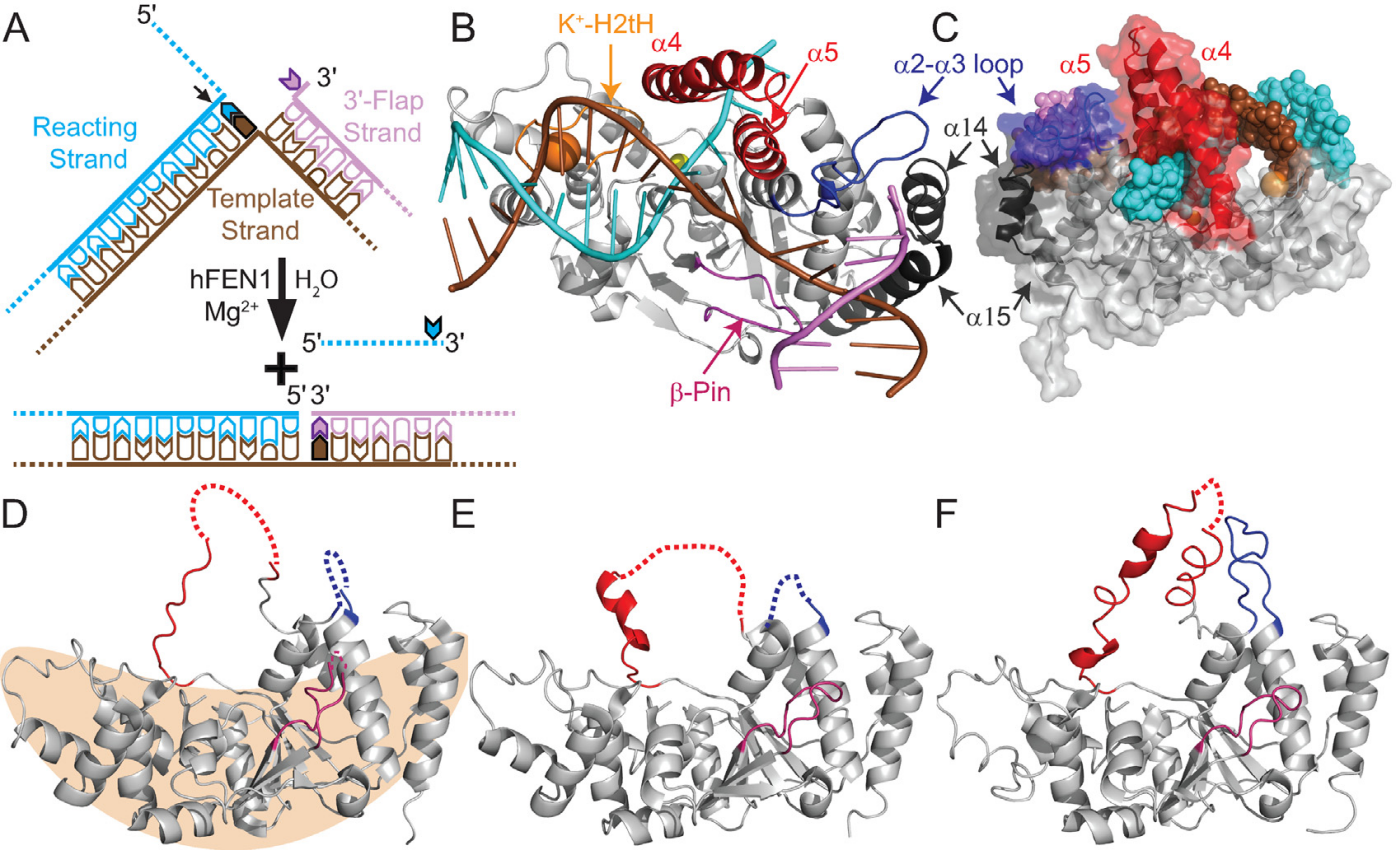
Regions of hFEN1 assume various conformations in crystal structures. (**A**) Representation of hFEN1-catalyzed reaction of double-flap DF#,1 creating ssDNA and nicked dsDNA products (2). # denotes a 5′-flap of any length, 1 denotes a single nucleotide 3′-flap. (**B**) The hFEN1_D86N_-(DF2,1) complex (5UM9) (21) shows that the arch (α4-α5, red), α2–α3 loop (blue) and β-pin (β6-β7 loop) (pink) are ordered in the presence of DNA substrate (colored as in panel A). The α10-α11 loop (H2tH motif) that binds the reacting duplex approximately 20 Å from the scissile phosphate and the α14-loop-α15 that is unique to FEN proteins are shown in orange and black, respectively. The potassium and active site samarium cations are shown as orange and yellow spheres, respectively. (**C**) Rear view of 5UM9 (cartoon with transparent surface representation) with regions colored as in panels A and B and the DNA (DF2,1) shown as spheres. (**D**−**F**) The three crystal structures of apo-hFEN1 (1UL1) show the arch, α2–α3 loop and β-pin regions in various states of order and disorder, dashed lines indicate missing electron density (22). (D) The tan shape illustrates the saddle-region of hFEN1 that binds the dsDNA and active site metals.

Flap endonuclease 1 (FEN1) is a member of the 5′-nuclease superfamily that is predominantly involved in Okazaki fragment maturation, but also has roles in long-patch base excision repair and ribonucleotide excision repair (1,2). All three of these pathways create bifurcated nucleic acid structures known as 5′-flaps that must be removed precisely to create single-stranded (ss) and nicked double-stranded (ds) DNA products (Figure 1A). In line with a critical role in DNA replication, FEN is present in all organisms, from bacteriophage to mammals (3). In humans, cancer cells overexpress FEN1, and tumor aggressiveness correlates with FEN1 protein levels (4). As such, human FEN1 (hFEN1) has been postulated to be a potential cancer therapeutic target (5,6), and evidence suggests that combinatorial targeting of hFEN1 has therapeutic relevance (7). Moreover, FEN1 is the prototypical member of the 5′-nuclease superfamily whose activities span all major DNA metabolic pathways. As nucleases, specificity is paramount as unwanted hydrolysis of DNA or RNA can be deleterious; thus, how hFEN1 and paralogues achieve substrate and scissile phosphate diester specificity has been an area of considerable debate.

Earlier studies revealed that hFEN1 specificity arises from the recognition of three structural features of its substrate (Figure 1A) (2,8). Firstly, only junction dsDNAs can be bound stably by the protein as two juxtaposed dsDNA binding sites require the substrate to bend roughly 100° (Figure 1B) (9–11). Secondly, despite removing 5′-flaps, hFEN1 recognizes substrates bearing a one nucleotide (1-nt) 3′-flap (12–14). In structures of hFEN1–DNA complexes, the 3′-flap is cradled in a pocket created from the α14–α15 loop, the first turn of α15 and the α2–α3 loop (11). The α2–α3 loop folds into a non-regular secondary structure known as an Ω-loop (15), often associated with allosterically-regulated regions of proteins (Supplementary Figure S1) (16). Thirdly, the 5′-flap portion of the substrate must pass under the arch region (α4-α5) for biologically-relevant rates of reaction to occur (Figure 1B and C) (17,18). This latter substrate selection feature is also shared by the 5′-nuclease superfamily member human exonuclease-1 (19,20), but not by other family members that act on continuous DNAs like bubble-cutting XPG and the Holliday junction resolvase GEN1 (3). When these three substrate recognition tests are met, the DNA can untwist and enter the active site for catalysis. Of the three hFEN1 substrate feature selection processes, the threading of the 5′-flap through the arch remains the most enigmatic.

Crystal structures of hFEN1–DNA complexes (3Q8K, 3Q8L, 3Q8M, 5UM9) show the arch is composed of two α-helices above the active site (11,21). Together these create a hole in the protein that is large enough to accommodate ssDNA but not dsDNA (11,21), which could explain hFEN1 specificity for 5′-ss flaps (Figure 1C). However, this is inconsistent with the ability of hFEN1 to process 5′-flaps with some duplex secondary structure (12,18). A further puzzle relates to how threading could occur through a small hole in the absence of an energy source. In contrast, apo-hFEN1 structures show that the regions corresponding to the arch and α2–α3 loop sometimes lack discernible electron density; what has been observed is either random coil or a limited degree of secondary structure (Figure 1D–F) with high B-factors (Supplementary Figure S2) (22). Furthermore, structures of FEN proteins from a wide array of organisms show the arch region in various conformations and positions (Supplementary Figure S3) (2). It is possible that structural heterogeneity of the arch and α2–α3 loop has some functional relevance. However, direct confirmation of the structural status of the arch in free and substrate bound hFEN1 is necessary to define its mechanism.

To extend our understanding, multidimensional NMR spectroscopy was used to assess the properties and dynamics of hFEN1 alone and in complex with DNA in solution. We show that the solution conformation of hFEN1 is consistent with the crystal structure in the dsDNA binding region known as the saddle (Figure 1D), but a large discrepancy is observed in the arch region (traditionally referred to as the helical arch). Using a combination of kinetic, spin relaxation, chemical shift and single molecule FRET data, the contribution of hFEN1 conformationally dynamic regions to substrate specificity is revealed. Chemical shift perturbation mapping provides evidence that divalent cations induce conformational changes in the hFEN1–DNA complex that are not observed in hFEN1 alone. These data demonstrate that the active site communicates with regions involved in dsDNA binding and substrate feature recognition. Combined data raise the possibility that both conformational selection and induced fit mechanisms occur in the arch region in response to structural features of the substrate and reveal that the hFEN1–DNA complex exhibits millisecond timescale motions in the arch. Taken together, our analysis shows that regional conformational flexibility spanning a range of dynamic timescales is channeled into the catalytically-competent conformational ensemble in response to substrate features, providing the required link between specificity and catalysis in a structure-sensing nuclease. Because the structures of other 5′-nuclease superfamily show disorder or indicate flexibility in the area that corresponds to the hFEN1 arch region (19,20,23–25), a similar conformational ensemble shift mechanism in response to other paralogue-specific triggers is likely important in their mechanisms as well.

## MATERIALS AND METHODS

### Protein expression

Human hFEN1-336 was expressed from *Escherichia coli* BL21(DE3)-RILP cells transformed with pET29b-hFEN1-336 plasmid, encoding for hFEN1-336 (herein referred to as hFEN1) protein containing a human rhinovirus (HRV) type 14 3C protease-cleavable (His)_6_-tag located at the C-terminus. Natural abundance protein for kinetic analyses was expressed and purified as described previously (7). ^15^N-labeled protein was expressed using ^15^N autoinduction media (26). The final concentrations of ^15^N autoinduction media components were: 50 mM Na_2_HPO_4_ and 50 mM KH_2_PO_4_ pH 7.5, 50 mM ^15^NH_4_Cl, 5 mM Na_2_SO_4_, 2 mM MgSO_4_, 0.5% glycerol, 0.05% d-glucose, 0.2% α-lactose, 12× BME vitamins (USBiological B0110), 1× Trace Metals (Teknova 1000x Trace Metals T1001), 400 μg ml^−1^ kanamycin and 34 μg ml^−1^ chloramphenicol. Cultures for ^15^N-labeled protein expression were inoculated with a 100-fold dilution of a saturated 2× YT starter culture, and were then allowed to grow at 37°C for 4 h before the temperature was reduced to 18°C to allow for overnight expression by autoinduction.

^2^H,^13^C,^15^N-labeled protein was prepared using a high cell-density procedure in combination with isopropyl-β-d-thiogalatopyranoside (IPTG) induction (27). The final concentrations of all media components were: 50 mM Na_2_HPO_4_ and 25 mM KH_2_PO_4_ pH 7.5, 18 mM ^15^NH_4_Cl, 1% ^13^C_6_,^2^H_7_-d-glucose (U-^13^C_6_, 99%; 1,2,3,4,5,6,6-d_7_ 97-98%), 0.2 mM CaCl_2_, 5 mM MgSO_4_, 10 mM NaCl, 0.25 × BME vitamins (USBiological B0110), 0.25 × Trace Metals (Teknova 1000x Trace Metals T1001), 400 μg.ml^−1^ kanamycin and 34 μg ml^−1^ chloramphenicol. Initially, a 100-fold dilution of a saturated culture of BL21(DE3)-RILP cells transformed with pET29b-hFEN1-336 was used to inoculate 2× YT media prepared with 50% D_2_O and was grown until saturation. A 100-fold dilution of this saturated 50% D_2_O 2× YT culture was then used to inoculate 2× YT media in 100% D_2_O to allow the cells to acclimatize for growth. Aliquots (50 ml) of these D_2_O acclimatized BL21(DE3)-RILP cells were added to 1 L of 100% D_2_O 2× YT media and were grown at 37°C until the *A*_600_ = 5–6. The culture was pelleted by ultracentrifugation (6000 × *g* at 4°C for 30 min) and the supernatant was discarded. The pellet was then resuspended in 1 L of ^15^N,^2^H,^13^C minimal media (as described above) and then allowed to grow at 37°C for a further 2 h to clear unlabeled metabolites. Afterward, IPTG was added to a final concentration of 0.1 mM IPTG, the temperature was reduced to 18°C, and the culture was grown for a further 72 h until *A*_600_ = 10–13. ^2^H,^15^N-labeled protein was prepared analogously except that the 1% ^13^C_6_,^2^H_7_-d-glucose (U-^13^C_6_, 99%; 1,2,3,4,5,6,6-d_7_ 97–98%) was replaced with 1% ^2^H_7_-d-glucose (1,2,3,4,5,6,6-d_7_ 97–98%).

In all cases, cells were harvested by centrifugation (6000 × *g* at 4°C for 15 min) and the supernatant was removed. The cell pellet was resuspended in 50 ml of ice-cold phosphate buffered saline (10 mM Na_2_HPO_4_ and 2 mM KH_2_PO_4_ pH 7.4, 137 mM NaCl, 2.7 mM KCl), the cells were pelleted again at (6000 × *g* at 4°C for 15 min) and the supernatant was removed. The cells were then resuspended in 50 ml of Buffer A (20 mM Tris–HCl pH 7.4, 1 M NaCl, 5 mM imidazole) containing 5 mM β-mercaptoethanol (βME), 0.1 mg ml^−1^ lysozyme and 1 EDTA-free protease inhibitor cocktail tablet (Sigma S8830). Cells were incubated on ice for at least 1 h and were frozen at −20°C until required.

### Protein purification

To purify hFEN1 protein, cells were thawed and then sonicated on ice for 60 cycles of pulsation for 5 s with 25 s cooling intervals. To each lysate, 5 ml of Buffer A containing 1% Tween-20 was added, and the lysate was clarified by centrifugation (40 000 × *g* at 4°C for 30 min). The supernatant was applied to a column (ID = 1.6 cm, length = 12 cm) containing Chelating Sepharose 6 Fast Flow agarose beads (GE Healthcare Life Sciences) that had been previously charged with Ni^2+^ ions according to the manufacturer's protocol and pre-equilibrated with 5 column volumes of Buffer A. The column was washed with 5 column volumes of Buffer A and 5 column volumes of Buffer B (25 mM Tris–HCl pH 7.4, 0.5 M NaCl, 40 mM imidazole, 0.01% Tween-20, 5 mM βME). The bound hFEN1 protein was eluted with 8 column volumes of Buffer C (250 mM imidazole pH 7.2, 0.5 M NaCl, 5 mM βME). Fractions containing hFEN1 protein were identified by SDS-PAGE, pooled and immediately diluted with an equal volume of Buffer D (20 mM Tris–HCl pH 7.4, 1 mM EDTA, 20 mM βME). The solution was then applied to a 5 mL HiTrap Q HP column (GE Healthcare Life Sciences) pre-equilibrated with the buffer D. hFEN1 protein was found in the flow through, whereas nucleic acid contamination was retained by the column and eluted using 10 column volumes of Buffer D containing a linear gradient of 0–1 M NaCl. The amount of hFEN1 protein in the flow through was estimated by measuring the absorbance at 280 nm (ϵ_280_ = 22 920 M^−1^ cm^−1^) (28) using a UV-Vis NanoDrop spectrophotometer (ThermoFisher Scientific). Two units of Turbo3C (HRV3C protease) (BioVision) were added for every mg of protein, and the mixture was allowed to stand overnight at 4°C to catalyze cleavage of the (His)_6_-tag from the hFEN1 protein. The extent of affinity tag removal was assessed by SDS-PAGE, and the solution was diluted further with an equal volume of buffer E (25 mM Tris–HCl pH 7.5, 1 mM CaCl_2_, 20 mM βME). The hFEN1 protein solution was then applied to a 20 ml HiPrep Heparin FF 16/10 column (GE Healthcare Life Sciences) pre-equilibrated with 2 column volumes of buffer E and eluted using 15 column volumes of Buffer E containing a linear gradient of 0–1 M NaCl (hFEN1 protein eluted at 350 mM NaCl). Fractions containing hFEN1 protein were pooled and concentrated to less than 10 ml using an Amicon Ultrafiltration cell with a 10 kDa MWCO PES membrane (MerckMillipore) pressurised using nitrogen gas. The protein was exchanged into the appropriate buffer (see below) using a HiPrep 26/10 desalting column (GE Healthcare Life Sciences). The hFEN1 protein concentration was determined using the absorbance at 280 nm as described above. Unlabeled samples for kinetic measurements were exchanged into 100 mM HEPES–KOH pH 7.5, 200 mM KCl, 1 mM EDTA, 10 mM DTT and 0.04% NaN_3_, and adjusted to a concentration of 200 μM. An equal volume of glycerol was added to each sample to allow optimal storage conditions (final concentration: 100 μM hFEN1, 50 mM HEPES–KOH pH 7.5, 100 mM KCl, 0.5 mM EDTA, 5 mM DTT, 0.02% NaN_3_ and 50% glycerol). Samples were stored at −20°C. Isotopically labeled hFEN1 protein samples were exchanged into the appropriate NMR buffer and concentrated to 0.5 mM using a Vivaspin 20 (10 kDa MWCO) concentrator (4000 × *g* at 4°C).

All purification buffers were filtered and degassed prior to use, and an ÄKTA pure chromatography system was used for all column purification steps. Unless otherwise stated, all reagents were purchased from Sigma-Aldrich, Fisher Scientific, or VWR International and used as received.

### NMR assignment

All NMR experiments were performed at 298 K (relative to d_4_-methanol signal) using standard pulse sequences on either a Bruker 600 MHz Avance DRX spectrometer equipped with a 5-mm TXI cryoprobe with z-axis gradients or a Bruker 800 MHz Avance I spectrometer equipped with a 5-mm TXI probe with z-axis gradients. Both spectrometers were operated with TopSpin 2. Comparison of ^1^H–^15^N TROSY spectra of ^2^H,^15^N,^13^C-labeled hFEN1 with ^15^N-labeled hFEN1 showed that all backbone amide groups had back exchanged from deuterium to protium atoms. For the backbone resonance assignment of hFEN1, 0.5 ml samples contained 0.5 mM ^2^H,^15^N,^13^C-labeled hFEN1 in 10 mM HEPES–KOH pH 7.5, 76 mM KCl, 0.1 mM EDTA, 4 mM NaN_3_, 100 mM βME. D_2_O (10% v/v) and 0.05 mM trimethylsilyl propanoic acid (TSP) were added for the deuterium lock and chemical shift reference standard, respectively. After transferring the sample into a 5 mm NMR tube, the sample was sealed with a Precision Seal^®^ rubber septa cap (Sigma-Aldrich Z554014) and argon was passed over the sample to help minimize oxidation.

hFEN1 backbone ^1^H^N^, ^15^N, ^13^C^α^, ^13^C^β^ and ^13^C' were assigned using the standard suite of ^1^H–^15^N TROSY and 3D TROSY-based constant time experiments (HNCA, HN(CO)CA, HNCACB, HN(CO)CACB, HN(CA)CO and HNCO) (29). ^1^H chemical shifts were referenced relative to the internal TSP signal, whereas ^15^N and ^13^C chemical shifts were indirectly referenced using nuclei-specific gyromagnetic ratios. Peak picking and frequency matching was performed within CCPNMR Analysis version 2.4 (30), and the backbone assignments were confirmed independently using a simulated annealing algorithm employed by the *asstools* assignment program (31). The secondary structure content and Random Coil Index-S^2^ (RCI-S^2^) prediction of hFEN1 was conducted by uploading the backbone ^1^H^N^, ^15^N, ^13^C^α^, ^13^C^β^ and ^13^C' chemical shifts to the TALOS-N webserver (32).

### NMR relaxation measurements

hFEN1 samples for ^15^N backbone fast timescale relaxation measurements were performed using ^2^H,^15^N-labeled hFEN1 in a 5 mm Shigemi NMR microtube assembly matched with D_2_O. Sample conditions were 10 mM HEPES–KOH pH 7.5, 76 mM KCl, 4 mM NaN_3_ 100 mM βME, 10% D_2_O, 0.05 mM TSP and 0.1 mM EDTA. Spin-lattice ^15^N relaxation rates (R_1_), rotating frame ^15^N relaxation rates (*R*_1ρ_) and heteronuclear steady-state ^15^N-{^1^H} NOE (hNOE) values were obtained using interleaved TROSY-readout pulse sequences (33). Temperature compensation was applied in the *R*_1_ experiment by incorporating a spin-lock pulse placed off-resonance in the inter-scan delay, equal to the longest spin-lock time and RF power of the *R*_1ρ_ experiment. Relaxation delays of 0, 80, 240, 400, 400, 640, 800, 1200, 1760 and 2400 ms were used to calculate *R*_1_, and delays of 1, 20, 20, 30, 40, 60, 90, 110, 150 and 200 ms were used to calculate R_1ρ_. The inter-scan delay was 3.5 s and the strength of the RF spin-lock field during *R*_1ρ_ measurement was 1400 Hz at 600 MHz and 1866.7 Hz at 800 MHz. For the hNOE measurement, two interleaved experiments were acquired with relaxation delays of 10 s. For the determination of *R*_1_ and *R*_1ρ_ rates, the decay of backbone amide peak intensities were fitted using an exponential function in CCPNMR (30). Relaxation parameters were obtained for 192 residues because 59 residues were omitted from further analysis due to peak overlap or poor signal to noise ratios. For hNOE experiments, the ratio of the saturated to unsaturated peak height was measured. *R*_2_ values were calculated from *R*_1_ and *R*_1ρ_ rates according to Equation ((1)).

(1) \begin{equation*}{{{R}}_2} = {{{R}}_{1{\rm{\rho }}}}/{\rm{si}}{{\rm{n}}^2}\theta - {{{R}}_1}/{\rm{ta}}{{\rm{n}}^2}\theta \end{equation*}

where tan *θ* = *ω*_1_ / Ω, *ω*_1_ is the spin lock RF field (1400 Hz at 600 MHz and 1866.7 Hz at 800 MHz) and Ω is the offset of the ^15^N resonance of interest with respect to the ^15^N carrier frequency (33).

### Model-free analysis

Model-free analysis was performed using *relax* (34–38) on the Sheffield-WRGRID ICEBERG high performance computing cluster. Using *R*_1_, *R*_2_ and hNOE values at 600 MHz and 800 MHz and the coordinate geometry of backbone amide N-H bonds as provided by the crystal structure 3Q8K (11), model-free analysis was executed. However, residues from the arch region, α2–α3 loop and the C-terminus were excluded due to lack of coordinates in the crystal structures (1UL1) (22) or low R_2_/R_1_ values. Instead, these 16 residues were modeled with a spherical diffusion tensor. Both analyses were conducted for the 192 residues for which relaxation data at both field strengths was available; of these, only 179 were processed fully, as 13 were removed due to large errors or computational eliminations.

### Titration of Ca^2+^ and Mg^2+^ cations into hFEN1_K93A_

A ^15^N-labeled hFEN1 K93A mutant (hFEN1_K93A_), which hindered catalysis, was prepared in the pET29b-hFEN1-336 vector as described previously (11). The hFEN1_K93A_ protein was prepared as described for the wild type above using the ^15^N autoinduction method. ^1^H–^15^N TROSY spectra were separately recorded at 10 mM HEPES–KOH pH 7.5, 76 mM KCl, 0.1 mM EDTA, 4 mM NaN_3_, 100 mM βME, 10% D_2_O and 0.05 mM TSP with either 0 mM, 8 mM MgCl_2_ and 8 mM CaCl_2_ added. Absolute changes in weighted chemical shifts (ω) were determined using Equation ((2)) where the correction factor for ^15^N was α = 0.14.

(2) \begin{equation*}{\rm{\omega }} = {\left( {\Delta {{\rm{\delta }}_{\rm{H}}}^2 + {{\left( {{\rm{\alpha \Delta }}{{\rm{\delta }}_{\rm{N}}}} \right)}^2}} \right)^{1/2}}\end{equation*}

### DNA preparation

DNA oligonucleotides were purchased with purification from LGC Biosearch Technologies. The sequence of the oligonucleotides are shown in Table 1. Oligonucleotide concentrations were quantified using absorbance at 260 nm and molar extinction coefficients calculated according to the ‘nearest-neighbor’ method (39,40).

**Table 1.** Oligonucleotide sequences used herein

Name	Sequence
*DHPS1*	5′dTGAAAGGCAGAGCGCTAGCTCTGCCTTTCGAGCGAAGCTCC3′
F1*	5′FAM*-dTTTTTACAAGGACTGCTCGACAC3′
*F1*	5′dTTTTTACAAGGACTGCTCGACAC3′
*T1*	5′dGTGTCGAGCAGTCCTTGTGACGACGAAGTCGTCC3′

*5′FAM is a 5′-terminal fluorescein modification produced using the single isomer 5-carboxyfluorescein-aminohexyl amidite.

### hFEN1_K93A_-DNA complex formation

DHPS1 (DNA substrate) was heated and annealed in 10 mM HEPES–KOH pH 7.5, 6 mM KCl, 4 mM NaN_3_ and 0.1 mM EDTA. Initially, 0.5 mM ^15^N-labeled hFEN1_K93A_, ^2^H,^13^C,^15^N-labeled hFEN1_K93A_ or ^2^H,^15^N-labeled hFEN1_K93A_, prepared as described above, in 10 mM HEPES–KOH pH 7.5, 76 mM KCl, 4 mM NaN_3_ and 0.1 mM EDTA was titrated with sub-stoichiometric aliquots of lyophilized DHPS1 until the DNA was in excess of the protein (>500 μM). At 50–100 μM of DNA, the sample precipitated and the quality of the spectra decreased rapidly. To overcome protein precipitating in complex with DNA, an hFEN1_K93A_-DNA complex was prepared in 10 mM HEPES–KOH pH 7.5, 6 mM KCl, 4 mM NaN_3_ and 0.1 mM EDTA in the presence of excess DNA substrate (1:1.1) under dilute conditions of protein and DNA (roughly 5 μM). Low monovalent salt concentrations were used to slow dissociation of the complex. The sample was concentrated to ∼500 μM using Vivaspin 20 (10 kDa MWCO) spin columns and 100 mM βME, 10% D_2_O (10% v/v) and 0.05 mM TSP were added. The same resonance assignment strategy using 3D NMR spectra was conducted with a ^2^H,^15^N,^13^C-labeled hFEN1_K93A_–DNA complex prepared in this manner. ^1^H-^15^N TROSY spectra were acquired at 0, 0.5, 1, 2, 4, 6 and 8 mM CaCl_2_, and chemical shift differences were recorded as described in Equation (2).

### Preparation of hFEN1 cysteine mutants labeled with fluorophores

Surface residues C235 and C311 were successively mutated to alanine in the pET28b-hFEN1 plasmid using Agilent site-directed mutagenesis protocols (41) and the appropriate oligonucleotides (Supplementary Table S1). The resulting double mutant hFEN1 protein (C235A/C311A) was expressed and purified as described above. This mutant did not react with maleimide dyes on a timescale of 1 h. Subsequent mutagenesis as above created hFEN1_Q_, E120C/C235A/S293C/C311A. hFEN1_Q_ protein was expressed and purified as described above. ESI-MS calculated: 43210.6 Da experimental: 43210.9 Da.

To stochastically label hFEN1_Q_ with the appropriate fluorophores, 250 μl of 100 μM (25 nmol) purified hFEN1_Q_ in 50% glycerol storage buffer was diluted 1:1 with buffer F (50 mM Tris–HCl pH_25°C_ 7, 150 mM NaCl, 1 mM EDTA and 0.1 mM TCEP) to dilute the glycerol. DTT was added to the diluted hFEN1_Q_ to a final concentration of 20 mM, and the solution allowed to incubate on ice for 15 min. The hFEN1_Q_ solution was then subjected to size exclusion chromatography (SEC) using a Superdex 75 GL 10/300 column (GE Healthcare Life Sciences) and buffer F. Protein containing fractions were pooled and concentrated using a 10 kDa MWCO Vivaspin-20. The hFEN1_Q_ protein concentration was quantified using the absorbance at *A*_280_ nm and calculated extinction coefficient (28). Stocks of 10 mM Atto 647N maleimide (Sigma-Aldrich) and 10 mM Cy3b maleimide (GE Healthcare Life Sciences) were prepared in DMSO. To label hFEN1_Q_, the protein was added to a tube containing the appropriate volume of each stock of fluorophore maleimide conjugate to give a molar ratio of 1:2:2 of hFEN1:Atto 647N:Cy3b. Aliquots (usually 50 μl) of the reaction were removed at 2, 4, 8, 16 and 26 min and quenched by addition of excess DTT. To remove excess free fluorophore, the quenched aliquots were exchanged into buffer F using micro Bio-Spin 6 columns (BioRad) according to the manufacturer's instructions. The labeled protein was again subjected to SEC as described above. The protein containing fractions were pooled and concentrated as before. The concentrations of the protein and the covalently attached Cy3b and Atto 647N were quantified using a UV/Vis Nanodrop spectrohphotometer (ThermoFisher) at *A*_280_, *A*_559_ and *A*_646_, respectively, and the associated extinction coefficients (hFEN1 protein ϵ_280_ = 22 920 M^−1^ cm^−1^, Cy3b ϵ_559_ = 130 000 M^−1^ cm^−1^ and Atto 647N ϵ_646_ = 150 000 M^−1^ cm^−1^). A_280_ values were corrected for contributions from both dyes using *A*_280_/*A*_556_ and *A*_280_/*A*_646_ ratios.

### Kinetic measurements of hFEN1, mutants and fluorescently labeled proteins

The activities of hFEN1, hFEN1_Q_ and hFEN1_Q_ with attached fluorophores (hFEN1_QF_) were assessed using a 5′-fluorescein (FAM) labeled bimolecular DNA oligo construct DF5,1*, which was prepared by heating and annealing F1* and T1 in a 1:1.1 ratio (Table 1), respectively, in 50 mM HEPES–KOH pH 7.5, 100 mM KCl and 0.02% NaN_3_. Reactions mixtures (180 μl) containing 50 nM DF5,1* in reaction buffer (55 mM HEPES–KOH pH 7.5, 110 mM KCl, 8 mM MgCl_2_, 0.1 mg ml^-1^ BSA. 1 mM DTT) were initiated by adding appropriate amounts of hFEN1 proteins (3–6 pM final concentration) that afforded less than ∼10% product formation in 10 min at 37°C. Aliquots (20 μl) of reaction were quenched in excess EDTA (50 μl of 500 mM EDTA) at 2, 4, 6, 8, 10 and 20 min. The amount of product formation was assessed by denaturing high performance liquid chromatography (dHPLC) as described previously (18,42). Plots of concentration of product versus time yielded the initial rate of reaction (ν_0_ nM min^−1^), which could be normalized for enzyme concentration (*ν*_0_/[E]_0_ min^−1^).

For second order rate constant determination as a function of viscosity, reaction mixtures (180 μl) were assayed at 2.5, 5, 7.5 and 10 nM DF5,1* substrate in reaction buffer at 37°C and the indicated relative viscosity. Buffer viscosity was adjusted using either glycerol (0−36% v/v) or sucrose (0−35%). Glycerol relative viscosities and their corresponding %v/v were calculated using temperature corrected density calculations at 37°C (43). Relative viscosity was adjusted with sucrose as described previously (42). Product formation was monitored by dHPLC and normalized initial rates of reaction were generated as described above. Estimates of second order rate constants were derived from the slope of normalized initial rate (*v*/[E]) versus the concentration of substrate [S]. A calculated second order rate constant (*k*_cat_/*K*_M_)_0_ value at relative viscosity of 1 was derived from the Y-intercept from a plot of *k*_cat_/*K*_M_ versus relative viscosity (44).

### Single molecule FRET measurements and analysis

Labeled hFEN1 (hFEN1_QF_) was diluted to ∼50 pM in binding buffer (55 mM HEPES–KOH pH 7.5, 110 mM KCl, 8 mM CaCl_2_, 0.1 mg ml^-1^ BSA, 1 mM DTT)) and incubated at room temperature for 5 min with or without 20 nM substrate DNA (i.e. DF5,1, prepared by heating and annealing F1 and T1 in a 1:1.1 ratio (Table 1)). Glass slides were passivated with 1 mg/ml BSA for 5 min prior to each measurement. Three triplicate 10-min data sets were acquired for each sample giving a total of 90 min of data for each condition, yielding ∼4000 bursts.

smFRET data were acquired using a custom built confocal microscope and alternating laser excitation (45). Two dioide lasers (515 nm and 635 nm – LuxX plus) were directly modulated (100 us, duty cycle 45%) and combined into an optical fibre. The output beam was collimated and then cropped to 2.5 mm diameter by an iris. The beam was directed into the back of the objective (Olympus UPLSAPO 60× NA = 1.35 oil immersion) using a dichroic mirror (Chroma ZT532/640 rpc 3 mm) with the fluorescence emission collected by the same objective, focussed onto a 20 um pinhole and then split (dichroic mirror: Chroma NC395323 – T640lpxr) for detection by two avalanche photodiodes (SPCM-AQRH-14 and SPCM-NIR-14, Excilitas). Photon arrival times were recorded by a national instruments card (PCIe-6353), with the acquisition controlled using custom software (LabView 7.1).

smFRET data analysis was performed using custom Jupyter notebooks, based on the open source python software, FRETBusts, described previously (46). Briefly, background rates in each channel were obtain for each 60 seconds of the acquisition by fitting a poisson distribution to inter-photon delays. This background rate was recalculated every 60 s to take account of any change in background throughout the measurement time. Bursts were identified using a dual channel burst search as previously described (47).

Apparent FRET efficiencies (E*) and apparent stoichiometries (S*) were calculated for each burst according to Equations (3) and (4), respectively:

(3) \begin{equation*}{E^*} = \frac{{{I_{A|D}}}}{{{I_{D|D}} + {I_{A|D}}}}\end{equation*}

(4) \begin{equation*}{S^*} = \frac{{{I_{D|D}} + {I_{A|D}}}}{{{I_{D|D}} + {I_{A|D}} + {I_{A|A}}}}\end{equation*}

where *I* represents the background corrected intensity in the (i) acceptor emission channel after donor excitation (*I*_*A*|*D*_), (ii) donor emission channel after donor excitation (*I*_*D*|*D*_) and (iii) acceptor emission channel after acceptor excitation (*I*_*A*|*A*_). Apparent FRET efficiencies were corrected for donor leakage, acceptor direct excitation, and the detection efficiencies/quantum yields (gamma correction) according to published protocols (48) (arXiv:1710.03807), using labeled DNA standards for the gamma correction.

After correction factors were applied, bursts were selected with at least 40 photons under green excitation, and 10 photons under red excitation, with a maximum of 300 photons to filter out an acceptor heavy aggregate observed in the data set. Bursts with an observed stoichiometry between 0.5 and 0.85 were plotted to generate a histogram of relative frequency versus FRET efficiency and fitted with an unrestrained double Gaussian function.

## RESULTS

### NMR assignments are consistent with known secondary structure and peak intensities suggest regions with unusual dynamic properties

**Figure 2.**
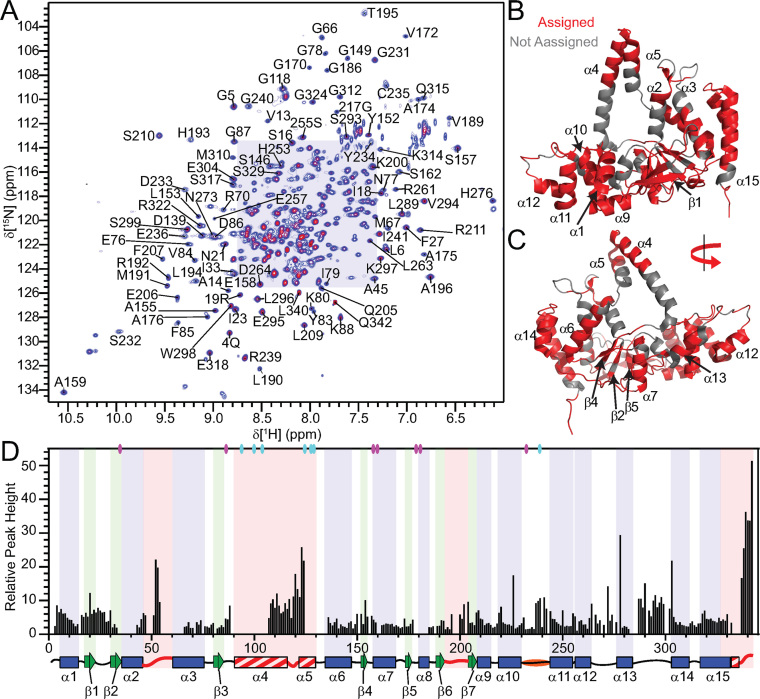
Relative peak heights suggest differences in dynamics in regions of hFEN1. (**A**) ^1^H–^15^N-TROSY spectrum of hFEN1 under experimental conditions. Peaks from amide side chains and the two tryptophan side-chain indole groups (H^ϵ^–N^ϵ^) are also observed. Expanded views of the shaded region can be found in Supplementary Figure S4. (**B**) Front and (**C**) rear views of the hFEN1 structure (3Q8K) (11) with labeled secondary structure elements and colored backbone to denote assigned (red) or unassigned (gray) residues. (**D**) Relative peak height (based on the lowest intensity peak E257) obtained from the ^1^H–^15^N-TROSY spectrum of hFEN1 plotted versus residue number show that loops are generally more intense and are flanked by decreasing peak intensities and sometimes missing residues. A secondary structure schematic of hFEN1 (3Q8K) is included (11). Blue rectangles, green arrows and black lines indicate α-helices, β-strands and loops, respectively. Loops known to have structural heterogeneity (1UL1 versus 3Q8K) are indicated by red lines (11,22). Red and white stripped rectangles highlight regions of structural heterogeneity. The H2tH α10–α11 loop is highlighted in orange. The location of the active site carboxylate (D34, D86, E158, E160, D179, D181 and D233) and basic residues (K93, R100, R104, K125, K128, R129 and R238) with respect to secondary structure elements are indicated by magenta and cyan ovals, respectively.

Human FEN1 (hFEN1) is a 380 amino acid protein that has a nuclease core domain (amino acid residues 1–336) that is sufficient for catalysis *in vitro*. As we were most interested in the dynamics associated with catalysis, the hFEN1–336 construct from X-ray crystallographic studies was used for NMR studies (11,21). Thus, the final 38 kDa hFEN1-336 construct (herein referred to as hFEN1) contains 336 native amino acids with an additional six residues present at the C-terminus from the rhinovirus 3C protease recognition site (49). Optimization of both buffer and temperature yielded stable hFEN1 samples that afforded good ^1^H–^15^N TROSY spectra (Figure 2A and Supplementary Figure S4). Using standard ^1^H–^15^N TROSY and TROSY-based 3D experiments (29), 251 amino acid residues out of the 324 theoretically-detectable backbone amides (excluding prolines and initiator methionine) were assigned in the absence of divalent metal ions. Many of the unassigned residues were localized to the base of the arch region, the active site amino acids and some of the α2–α3 loop (Figure 2B and C). Furthermore, the peaks in the ^1^H–^15^N TROSY spectrum displayed variable intensities, suggesting that not all residues relaxed uniformly. Some of the interfaces between the assignable and unassignable regions had peaks with decreased intensity, which suggested that the missing regions were undergoing exchange on the millisecond timescale (Figure 2D).

Chemical shift analysis of protein backbone nuclei (^15^N, ^1^H^N^, ^13^C^α^, ^13^C^β^ and ^13^C^'^) was conducted using TALOS-N (32). The predicted secondary structure elements agreed with the structure of the saddle region of the protein in 3Q8K and 1UL1x, thereby supporting the accuracy of our assignments (Supplementary Figure S5). Prediction of β-strand ϕ and ψ dihedral angles for residues E295−S299 were confirmed with further analysis of this region in 3Q8K and 1UL1z (11,22), which showed that the dihedral angles were consistent with the β-strand prediction despite being a loop. Furthermore, a single α-helical turn in the β1–β2 loop (residues D22−Y26) is present consistent with the TALOS-N assignment (11,22). In the arch region, the analysis for A106−A111 suggested that these residues were in an α-helical conformation. For Q112−Q115, α-helix was also predicted, but with decreasing probability. Finally, an extended loop conformation was predicted for residues A116−E124. Therefore, only the N-terminal half of the arch region was consistent with the secondary structure present in 3Q8K, whereas the C-terminal portion was not.

### The observable residues of the arch region and α2–α3 loop are disordered

To characterize the dynamics of the free protein, ^15^N spin-lattice (R_1_) and spin-spin (R_2_) relaxation rates and ^15^N-{^1^H} heteroNOE (hNOE) values were measured using ^2^H,^15^N-labeled hFEN1 (33). Of the 251 residues that were assigned, only 203 and 197 were analyzed at 600 and 800 MHz (Supplementary Figure S6A-F), respectively, due to peaks either being too weak to analyze or overlapped. The data generally showed that residues in secondary structure have similar relaxation rates, indicative of the overall rotational correlation time of the molecule (τ_c_). Using *R*_2_/*R*_1_ ratios (Supplementary Figure S6G and H), an overall τ_c_ was estimated to be 25 ns (50), consistent for a protein of this size (38 kDa) (51). Loops including β6–β7 (i.e. β-pin), α10–α11, α12–α13 and α13–α14 as well as the start of α15 displayed intermediate R_2_/R_1_ ratios. Regions that showed particularly low *R*_2_/*R*_1_ ratios and low hNOE values were the α2–α3 loop, the arch region and the non-native C-terminal residues. These regions were associated with higher than average *B*-factors or lacked observable electron density in crystal structures (Figure 1D–F and Supplementary Figure S2) (11,22). Furthermore, the higher peak intensities of the α2–α3 loop, the arch region and the non-native C-terminal residues (Figure 2D) are consistent with the lower *R*_2_/*R*_1_ ratios (Supplementary Figure S6G and H).

**Figure 3.**
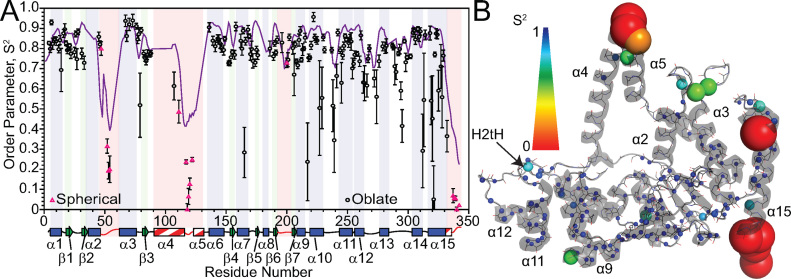
Model-free analysis of hFEN1 relaxation data identifies regions with increased flexibility. (**A**) Generalized order parameters (*S*^2^) were derived from *relax* (52) using backbone ^15^N relaxation data acquired at two field strengths and plotted versus residue number. Black circles represent data fitted to the oblate spheroid diffusion tensor, whereas pink triangles were fitted to a spherical diffusion tensor. The purple line in (**A**) represents the TALOS-N (32) random coil index *S*^2^ prediction. Secondary structure map as described in Figure 2D. (**B**) *S*^2^ values plotted on a cartoon depiction of the hFEN1 protein structure (3Q8K) (11). The spheres represent the nitrogen nuclei for which data were derived. The *S*^2^ and *R*_ex_ spectrum bars illustrate the magnitude of *S*^2^ and *R*_ex_ values with respect to sphere color and size.

We used *relax* in combination with the 3Q8K protein structure to calculate model-free order parameters (52). Residues with low *R*_2_/*R*_1_ ratios and a lack of density in the free protein structures were treated separately as an isotropic spherical diffusion tensor (16 residues), while the other backbone amide residues were derived using an oblate rotational diffusion tensor (163 residues) (Supplementary Figure S6I and J). Although a large range of *S*^2^ values were generated with various models (Supplementary Table S2), most residues in the protein in secondary structure elements afforded an average *S*^2^ of ∼0.8, showing that relaxation of most residues in the saddle region of hFEN1 was influenced predominantly by the tumbling of the molecule in solution (Figure 3). Therefore, these residues were relatively rigid. The α10–α11, α12–α13 and α13–α14 loops, the C-terminal end of α10 and α14 as well as most of α15 displayed intermediate *S*^2^ (0.5 < *S*^2^ < 0.8) values indicating increased flexibility in these regions. The lowest *S*^2^ values (<0.5) were generally observed in the C-terminal tail, arch and α2–α3 loop residues, thereby indicating that these residues were extremely flexible in solution. *R*_ex_ terms were reported for approximately 70% of the residues in hFEN1 that were amenable to model-free analysis (Supplementary Figure S7), indicating that millisecond timescale motions contributed to spin-spin relaxation (*R*_2_). Most of the *R*_ex_ terms were associated with residues in the saddle region.

**Figure 4.**
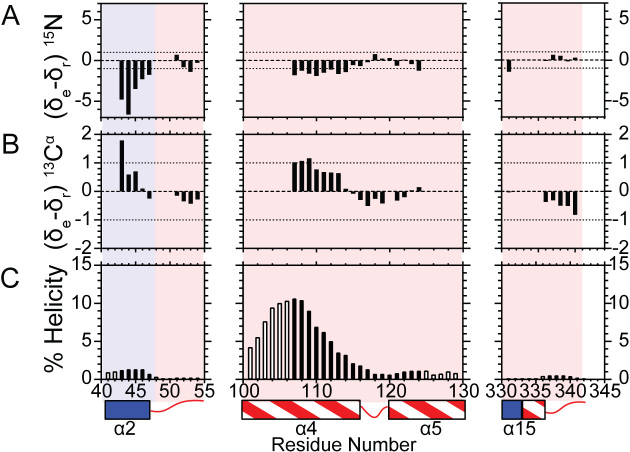
^15^N^H^ and ^13^C^α^ chemical shifts for the disordered arch region indicate that only some of the residues are sampling helical conformations. Bar graphs indicate the magnitude of the difference of experimental (δ_e_) and sequence-corrected random coil chemical shifts (δ_r_) for (**A**) ^15^N^H^ and (**B**) ^13^C^α^ for the indicated regions. Differences less than one (dotted line) suggest disorder. The anti-correlated nature of ^15^N^H^ and ^13^C^α^ is used to suggest which segments of the disordered region populate either helical or extended peptide backbone angles. Positive and negative ^15^N^H^ and ^13^C^α^ chemical shift differences, respectively, imply extended backbone angles, whereas negative and positive ^15^N^H^ and ^13^C^α^ chemical shift differences, respectively, suggest sampling α-helical backbone angles (53). (**C**) Agadir helical percentage predictions for the indicated residues assessed at 114 mM ionic strength, pH 7.5 and 25°C (NMR conditions) (54). Black bars indicate the residues for which NMR data is available to validate the prediction, whereas open bars indicate an absence of NMR assignments. A secondary structure map is illustrated below as described in Figure 2D.

*S* ^2^ values were compared with Random Coil Index-*S*^2^ (RCI-*S*^2^) values predicted by TALOS-N (32) and were congruent apart from an underestimation of the degree of regional flexibility (Figure 3A). Considering the flexibility of the arch, α2–α3 loop and C-terminal tail, we decided to confirm the nature of these regions using the neighbor-corrected random coil chemical shift library for intrinsically disordered proteins (ncIDP) (53). Deviations greater than 1 ppm from ncIDP ^15^N and ^13^C chemical shift values were shown to be a powerful indicator of secondary structure. The observable regions of the arch, α2–α3 loop and C-terminal residues showed only small differences (≤1) (Figure 4A and B), suggesting that these residues were disordered in the free protein. The small deviations from the random coil value suggested that residues A107−Q113 were transiently sampling α-helical ϕ,ψ-space. The postulated preference for occupying α-helical-ϕ,ψ space showed a gradual decrease occurring from Q110−A114. For residues A114−A120, a progressive switch to extended ϕ,ψ-space was implied (53). These data agreed with AGADIR predictions (54) and TALOS-N analysis (32) (Figure 4C and Supplementary Figure S5A). Overall, these data suggested that the middle of α4 was poised to form α-helical structure, whereas the top of α5 was not.

The analysis of the sequence of the arch residues (P90−L130) using CIDER (55,56) (Classification of Intrinsically Disordered Ensemble Regions) categorized this IDR as a polyampholytic coil or hairpin. Whether a polyampholytic IDR prefers to be a hairpin collapsed on itself or an extended coil (Supplementary Figure S3C,D,F) was shown to depend on the linear sequence distribution of charged residues, which can be assessed by the value κ (57). A κ value of 0 indicates no self-assembly, whereas a κ value of 1 indicates full collapse. The arch region has a very low κ value of 0.12, which suggested that it prefers to adopt an extended coil rather than collapsed conformation in solution.

### Divalent cations induce chemical shift changes in the vicinity of the hFEN1 active site

**Figure 5.**
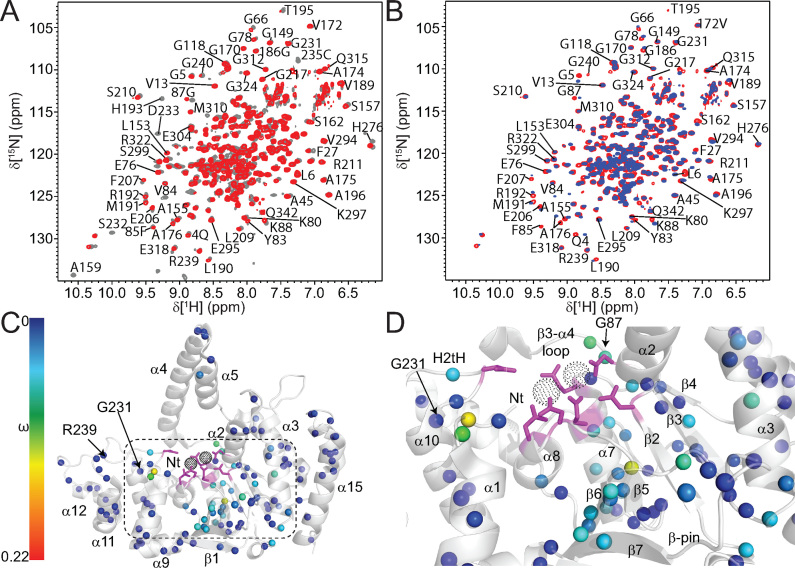
Addition of Ca^2+^ ions to hFEN1_K93A_ perturbs chemical shifts nearest the active site. (**A**) Superposed ^1^H–^15^N TROSY spectra of hFEN1 (gray) and hFEN1_K93A_ (red) shows only minimal changes in localized regions close to the mutation site indicating no widespread effects on global structure. (**B**) Superposed ^1^H-^15^N TROSY spectra of hFEN1_K93A_ in the absence (red) and presence (blue) of Ca^2+^. Well-dispersed residues are labeled accordingly. (**C**) Chemical shift changes observed on the addition of Ca^2+^ ions to hFEN1_K93A_ are mapped onto a cartoon representation of the hFEN1 structure (3Q8K) (11). The black-dotted spheres indicate the locations of active site metal ions that are coordinated by the side chains of carboxylate residues shown as magenta sticks. Secondary structure elements, the H2tH motif and the N-terminus (Nt) are labeled. The locations of the G231 and R239 spheres are highlighted. The magnitudes of the nitrogen nuclei chemical shift perturbations (ω) are represented by sphere color according to the associated spectrum bar. The absence of a sphere either indicates the lack of assignment in the protein or the inability to follow chemical shift changes due to peak overlap. (**D**) Magnified view of the area indicated by the dashed box in panel C to highlight the location of residues most affected by the addition of Ca^2+^ ions to hFEN1_K93A_. Labels are as described in panel C except for the omission of R239 and addition of G87.

Because hFEN1 binds two Mg^2+^ metal ions in the active site to catalyze the hydrolysis of the scissile phosphate diester bond of the optimal substrate (1,2), we wanted to assess the effects of Mg^2+^ and Ca^2+^ metal ion coordination by hFEN1 using chemical shift perturbation mapping. Ca^2+^ was included in the analysis for studies of an hFEN1–DNA complex, because it facilitates the hFEN1-induced DNA conformational change required for scissile phosphate diester placement on the active site metal ions without substrate hydrolysis (9,58). Furthermore, the analysis was performed using hFEN1_K93A_ because addition of Ca^2+^ alone was unable to completely inhibit phosphate diester hydrolysis, presumably due to small amounts of Mg^2+^ contamination (59). Removal of K93 by mutation to alanine reduces scissile phosphate diester hydrolysis by 2000-fold, because it acts as an electrophilic catalyst during scissile phosphate diester hydrolysis (42). Nonetheless, hFEN1_K93A_ still facilitates substrate conformational change similarly to hFEN1 (9,58). The K93A mutation is in a region of hFEN1 that was unable to be assigned. Comparison of the ^1^H–^15^N TROSY spectra of hFEN1 and hFEN1_K93A_ showed only small and localized changes in chemical shifts, thereby indicating that it had no widespread effect on the protein (Figure 5A).

Titration of either MgCl_2_ or CaCl_2_ (0–10 mM) into samples of hFEN1 was monitored using ^1^H–^15^N TROSY spectra and showed saturation between 8–10 mM for both divalent salts, consistent with the optimal concentrations of MgCl_2_ used in routine kinetic assays. Furthermore, ^1^H–^15^N TROSY spectra of hFEN1 with 8 mM Ca^2+^ or 8 mM Mg^2+^ are nearly identical, but show small differences in residues near to the active site including the N-terminal residues (Supplementary Figure S8A) This indicated that Ca^2+^ binds in the active site, consistent with Ca^2+^ being competitive with respect to Mg^2+^ (60). The small deviations in chemical shifts of residues closest to the active site could be due to the larger ionic radius and looser coordination geometry of Ca^2+^ compared to Mg^2+^. Comparison of ^1^H-^15^N TROSY spectra of hFEN1_K93A_ in the absence and presence of 8 mM Ca^2+^ showed similar chemical shift perturbations as observed in spectra of hFEN1 when Mg^2+^ or Ca^2+^ were added. The largest changes between apo-hFEN1_K93A_ and Ca^2+^-hFEN1_K93A_ occurred in regions close to the metal chelating carboxylates (D34, D86, E158, E160, D179, E181, D233) and the N-terminal residues (Figure 5B−D). Other regions affected included β5, α3, α7, β4–α7 loop, the H2tH motif, the β3–α4 loop and the β6-β7 β-pin, which are all regions that are near active site residues. Some of the β-strands also showed smaller perturbations further from the active site that suggested that conformational rearrangement in the active site is propagated throughout the β-sheet. These data indicated that divalent ions predominantly affected the structure immediately surrounding the active site in agreement with their locations in X-ray crystal structures (11). Notably, the addition of Ca^2+^ did not change the higher relative intensities of the peaks associated with the arch region and α2–α3 loop, suggesting that Ca^2+^ did not significantly alter the dynamics of these regions.

### Substrate binding changes the exchange regime for the arch region and α2–α3 loop

To prepare an hFEN1_K93A_–DNA complex and to analyze NMR spectral changes upon binding, consecutive sub-stoichiometric amounts of DNA substrate (DHPS1; Table 1 and Supplementary Figure S9A) were added to an hFEN1_K93A_ sample containing with 76 mM KCl until the DNA was in excess. However, addition of substrate induced protein precipitation in the sample suggesting non-specific DNA binding at concentrations used for NMR spectroscopy. Despite this, chemical shift changes upon addition of DNA were observed for a number of residues, consistent with locations known to interact with duplex DNA. Residues that were affected by DNA binding, but remained in the fast exchange regime (e.g. Q4, A175, L190, R192, R239 and G324) could be followed with the addition of DNA to saturation. Interestingly, the assignable α2–α3 loop and arch residues disappeared from the spectrum at sub-stoichiometric amounts of DNA, suggesting that they entered an exchange regime that prevented their detection (29). The magnitude of the exchange rate at which this happens at these field strengths should be faster than 10 s^−1^ but slower than 1000 s^−1^ as the peaks do not re-appear upon further addition of DNA to saturation. The switch from sub-nanosecond dynamics to millisecond exchange suggested a conformational change of the α2–α3 loop and the arch upon DNA binding.

### Divalent metal ions induce large chemical shift changes far from the active site

To overcome issues associated with the precipitation of hFEN1_K93A_ when preparing an hFEN1_K93A_–DNA complex (Supplementary Figure S9A), a different strategy was adopted where the complex was prepared under dilute conditions and at lower KCl concentrations in the presence of EDTA, followed by concentration. Preparing hFEN1_K93A_-DNA complexes in this manner resulted in stable samples that afforded acceptable ^1^H–^15^N TROSY spectra (Supplementary Figure S8B). Comparison of ^1^H–^15^N TROSY spectra of hFEN1_K93A_ and the hFEN1_K93A_-DNA complex showed chemical shift changes for residues in or near areas known to interact with the substrate, such as the H2tH motif (e.g. G231 and R239), active site (e.g. Q4, G5, L6, G87, K88, A175 and R192), the hydrophobic wedge (e.g. A45 and G66,) and the 3′-flap binding pocket (e.g. M310, E318 and G324). This indicated that the solution complex was consistent with the hFEN1–DNA complex crystal structures (3Q8K and 5UM9) (11,21). Before assigning the hFEN1_K93A_–DNA complex, Ca^2+^ was titrated into the sample (0–8 mM) and chemical shift perturbations upon each successive addition were monitored by ^1^H-^15^N TROSY spectra. Assignment of the hFEN1_K93A_-DNA complex in 8 mM Ca^2+^ was conducted using the same methodology as for hFEN1; however, only 57% of the visible peaks (132 peaks out 230 peaks in the spectrum) could be assigned presumably due to signal attenuation in the 3D spectra. Some assignments were corroborated by following the DNA titration data (i.e. R19, G149, A175, S255, R261).

**Figure 6.**
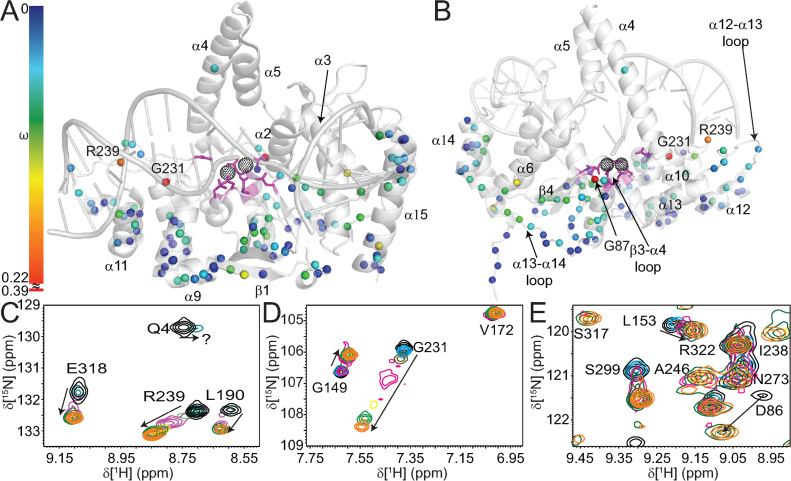
Addition of Ca^2+^ ions to the hFEN1_K93A_–DNA complex show chemical shift perturbations (ω) throughout the protein. (**A**) Front and (**B**) rear views of a cartoon depiction of the hFEN1–DNA structure (3Q8K) (11) with the magnitudes of nitrogen nuclei chemical shift perturbations (ω) on addition of Ca^2+^ represented by sphere color according to the associated spectrum bar. Note, because the chemical shift change for G231 was larger than for most other residues, the spectrum bar is discontinuous. (C−E) Highlighted regions from Supplementary Figure S8C showing several residues with significant chemical shift perturbations that occur upon titration with Ca^2+^ at 0 mM (black), 0.5 mM (cyan), 1 mM (olive), 2 mM (magenta), 4 mM (yellow), 6 mM (green) and 8 mM (orange). The panels show a large ω for (**C**) L190, R239 and E318, (**D**) G149 and G231 and (**E**) the appearance of S317 at higher Ca^2+^ concentrations. Intermediate exchange behavior is highlighted for (C) Q4 and (E) D86.

Addition of Ca^2+^ to hFEN1_K93A_ and the hFEN1_K93A_-DNA complex showed that residues immediately surrounding the active site were perturbed similarly (Figure 5B–D*cf*. Figure 6A and B), indicating that Ca^2+^ is coordinated in the active site in both free and bound forms. In the hFEN1_K93A_–DNA complex, however, the presence of Ca^2+^ additionally resulted in the disappearance of the N-terminal residues Q4, G5 and L6 (Supplementary Figure S8C). For example, the first addition of Ca^2+^ to the complex resulted in an upfield chemical shift with peak broadening for Q4 (Figure 6C). These N-terminal residues remained observable in spectra when Ca^2+^ was added to hFEN1_K93A_ (Figure 5B); thus, this unique effect was due to the presence of the DNA. Together this suggested that the addition of Ca^2+^ to the complex resulted in the N-terminal residues experiencing changes in their chemical environment due to movement of the residues themselves or movement of the DNA into the active site, thereby resulting in intermediate exchange broadening.

Unlike hFEN1_K93A_, the addition of Ca^2+^ to the hFEN1_K93A_–DNA complex resulted in larger perturbations in regions significantly removed from the active site (Figure 5C and D*cf*. Figure 6A and B). The most pronounced weighted ^1^H–^15^N chemical shift change (ω) was observed for G231, which lies at the beginning of the H2tH motif. In the absence of DNA, this residue showed a slight change in chemical shift upon addition of Ca^2+^ ions (ω = 0.022 ppm, Figure 5C), whereas the change increased greatly (ω = 0.39 ppm, Figure 6D) when DNA was present. Another H2tH motif residue whose Ca^2+^ induced chemical shift change was significantly larger for the hFEN1_K93A_–DNA complex was R239 (ω = 0.013 ppm, Figure 5B*cf*. ω = 0.17 ppm, Figure 6C), which lines the minor groove of the dsDNA in the reacting duplex. This showed that the H2tH DNA binding motif and residues in that region, which are approximately 20 Å from the active site metal ions, responded to the metal occupancy status of the active site in the presence of DNA. Residue G87, which is next to the divalent metal binding residue D86, also showed a larger chemical shift perturbation in response to Ca^2+^ in the hFEN1_K93A_-DNA complex (ω = 0.22 ppm, Figure 6B and Supplementary Figure S8C) compared to hFEN1_K93A_ (ω = 0.07 ppm, Figure 5B and Supplementary Figure S8B). In the threaded hFEN1–DNA substrate crystal structure (5UM9), residue G87, which is in the β3–α4 loop, is near the 5′-flap exit, and this larger perturbation was likely due to its proximity to the exit of the 5′-flap (Figure 1C) (21). Moreover, chemical shift changes in this region are consistent with the reported changes in the 5′-flap threading equilibrium when Ca^2+^ was added to hFEN1-DNA complexes (18). Furthermore, addition of Ca^2+^ to the hFEN1_K93A_–DNA complex also gave rise to larger chemical shift perturbations than in hFEN1_K93A_ in the β3–α4, α12–α13 and α13–α14 loops and helices α6, α14 and α15 (Figure 6A and B). These regions were proximal to the 5′-flap exit site and close to the 3′-flap binding pocket.

In contrast to the N-terminal residues, S317 of the hFEN1_K93A_–DNA complex reappeared near the end of the Ca^2+^ titration (Figure 6E). In the hFEN1–DNA product and substrate complexes (3Q8K and 5UM9) (11,21), the S317 backbone amide group hydrogen bonds with one of the non-bridging phosphate oxygen atoms of the 3′-flap phosphate diester moiety (Supplementary Figure S1A). In ^1^H–^15^N TROSY spectra of hFEN1_K93A_, S317 was observable in the presence and absence of Ca^2+^; however, the peak was missing from spectra of the hFEN1_K93A_-DNA complex in the absence of Ca^2+^ (Supplementary Figure S8B). The weighted chemical shift perturbation (ω) for S317 in hFEN1_K93A_ and the hFEN1_K93A_–DNA complex in the presence of Ca^2+^ was 0.68 ppm downfield, consistent with it forming a hydrogen bond with a non-bridging phosphate oxygen atom in the DNA. Regardless of the presence or absence of Ca^2+^, residues associated with the arch region and α2–α3 loop were neither observable nor assignable in spectra of the hFEN1_K93A_–DNA complex.

### Single molecule FRET measurements confirm conformational changes in the arch region

**Figure 7.**
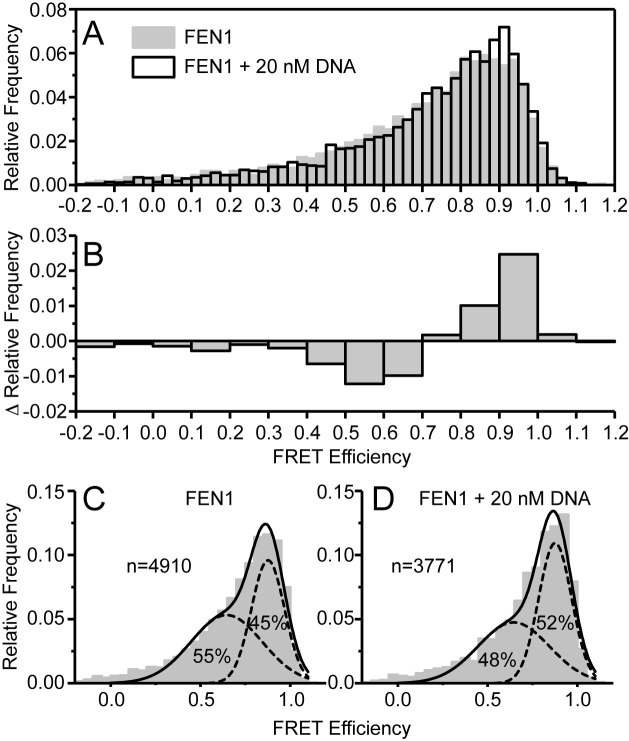
smFRET shows a change in conformational ensemble upon addition of DNA and reveals millisecond conformational dynamics occurring in the arch region. (A) Relative frequencies of FRET efficiencies for hFEN1_QF_ alone (gray) and in complex with 20 nM DNA (white). (**B**) The difference in relative frequencies of FRET efficiencies upon addition of 20nM DNA. (**C**) hFEN1_QF_ alone and (**D**) hFEN1_QF_ with 20 nM DNA showing the unrestrained fit to the sum of two Gaussian functions (see Supplementary Table S4 for fitting parameters) to show that upon addition of DNA the lower FRET population decreases and the higher FRET population increases.

To investigate further the range of conformations adopted by FEN1 in the presence and absence of substrate DNA (Supplementary Figure S9B without the 5′-FAM label), we used single-molecule Forster Resonance Energy transfer (smFRET). Modeling of dye attachment sites for donor (Cy3B) and acceptor (Atto647N) dyes identified sites in the arch (E120) and the saddle region (S293) opposite the DNA binding site that would produce a FRET pair sensitive to the conformation and position of the arch (Supplementary Figure S10A). However, hFEN1 has two partially solvent accessible cysteines (C235 and C311) that easily label with maleimide dyes (data not shown). Thus, a quadruple mutant was generated by successive site-directed mutagenesis (E120C/C235A/S293C/C311A; hFEN1_Q_) (41), and stochastic incorporation of the maleimide dyes was achieved at the desired postions to generate hFEN1_QF_ (Supplementary Table S3) (61). Measurement of hFEN1_Q_ and hFEN1_QF_ activities showed that the mutations and attachment of the dyes had only a small effect on enzymatic activity (Supplementary Figure S10B). In the absence of DNA but with Ca^2+^, a broad range of FRET efficiencies were observed (Figure 7A), indicative of multiple conformations and positions of the arch. In these experiments, single donor–acceptor labeled FEN1 molecules are observed as they diffuse through the confocal volume, a process which takes ∼ 1 ms. Conformations that interconvert much faster than the observation time yield a single, tight FRET efficiency signal, whereas a broad profile is indicative of multiple conformations converting on slower (> 1 ms) time scales, as was observed here (Figure 7A).

On addition of 20 nM substrate DNA, subtle changes in the FRET efficiency distribution were observed (Figure 7A); a decrease in the relative frequency of FRET efficiencies below 0.7 with a concomitant increase in FRET efficiencies above 0.7 (Figure 7B). This is indicative of a change in the conformational ensemble for FEN1 upon binding the DNA substrate. This change in the conformational ensemble could explain the unusual viscogen dependence of hFEN1 reaction (Supplementary Figure S11). The FRET efficiency distributions were well described by the sum of two gaussians, with free fits yielding the same means for the high and low FRET populations in both the presence and absence of the DNA substrate (Supplementary Table S4). On addition of DNA, a shift in the relative amplitude from the low-FRET to the high-FRET ensemble was observed (Figure 7C and D, Supplementary Table S4), showing a change in the conformational ensemble of the arch with respect to the saddle upon binding DNA. These data indicated that a change in the conformational ensemble in the arch occurred when substrate was added.

## DISCUSSION

**Figure 8.**
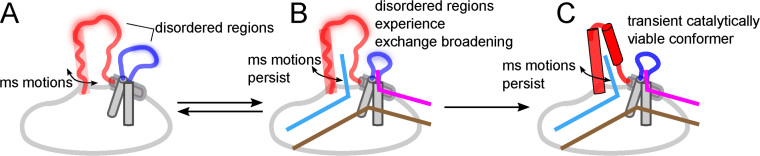
The flexible regions of hFEN1 respond to the appropriate structural features of the substrate, resulting in a shift to the catalytically viable ensemble. (**A**) In the absence of substrate, hFEN1 contains flexible regions in the ‘arch’ (red) and the α2–α3 loop (blue). The tops of these regions are disordered and display very fast motions (∼10^9^ s^−1^), whereas the flanking regions are absent presumably due to intermediate exchange broadening. The observable regions of the arch and loop are likely in fast exchange because they experience little to no change in chemical environment from the millisecond timescale motion. (**B**) Upon binding DNA, the disordered regions experience exchange broadening, likely due to a change in chemical environment and/or change in rate of motions. However, the slow millisecond timescale movements persist in both the arch and the α2–α3 loop despite the change in the conformational ensemble. (**C**) Eventually, the catalytically viable ensemble forms possibly coupling motions within the arch, the α2–α3 loop and the DNA.

Earlier studies have suggested that hFEN1-specificity for DNA structure rather than sequence arises from the recognition of three substrate structural features: (i) the bending of the two-way dsDNA junction, (ii) 5′-flap threading and (iii) 3′-flap binding (8–12,21). The latter two substrate-feature selection steps involve regions of the protein that we show are conformationally dynamic (i.e., the arch region and the α2–α3 loop). NMR spin relaxation data for the free protein shows that residues in the top of the arch (A107, L111, A117–E120 and E122) and the middle of the α2–α3 loop (V52–Q54) are very flexible, exhibiting motions on the timescale of ∼10^9^ s^−1^; hence, they are disordered. However, residues either side of these flexible regions cannot be detected in NMR experiments presumably due to intermediate exchange broadening caused by millisecond timescale motions. Consistent with this, smFRET data for the free protein suggests that the arch region is moving relative to the saddle region of hFEN1 at a rate slower than a 1000 s^−1^ (Figure 8). The highly mobile residues in the arch and α2–α3 loop that are observable by NMR may experience this millisecond motion as well, but because these regions likely experience negligible chemical shift changes, the sub-nanosecond timescale motions dominate the relaxation.

The observable residues in arch and α2–α3 loop undergo a change of motional timescale from ∼10^9^ s^−1^ in the free protein to an intermediate exchange regime (10–1000 s^−1^) in the hFEN1–DNA complex, suggesting their chemical environment is perturbed more significantly when bound to DNA. Such perturbation can arise from changes in rates of exchange, secondary structure formation, and changes in chemical environment due to proximity to the DNA. In line with this, the smFRET data indicate a change in the conformational ensemble upon addition of DNA. Interestingly, both the smFRET and NMR data suggest that there are still millisecond time motions involving the arch and α2–α3 loop. Thus, the earlier disorder to order model upon correct substrate binding that was based on crystallographic observations (2,11,21,62,63) is an oversimplification as it suggests there are no motions in the hFEN1–DNA complex. Rather, the complex is likely better described as an ensemble of conformations. The smFRET data shows that addition of DNA shifts the equilibrium bias of the arch to higher FRET efficiency states, implying the net position of the arch moves closer to the saddle. Interestingly, the arch has been observed in different positions relative to the saddle in hFEN1 (11,21) and hEXO1 X-ray structures (20,64). Notably, in structures where DNA has untwisted and moved into the active site (21), the arch has moved forward toward the saddle. It is possible that arch motions play an important role in the catalytic cycle, by facilitating untwisting of the DNA to transfer the scissile phosphate to the active site. Indeed, the single turnover rates of the hFEN1-catalyzed reaction are 15–30 s^−1^ (12); thus, it is possible that the slower conformational changes in the arch and α2–α3 loop regions could limit the rate of reaction.

In the hFEN1–DNA complex, residues near the active site are perturbed upon addition of Ca^2+^ as is also seen with substrate-free hFEN1. Interestingly, the N-terminal residues disappear from the spectra upon addition of Ca^2+^ to hFEN1–DNA, but not with hFEN1 alone. The recent crystal structures of the hFEN1–DNA complex (5UM9) suggests that the N-terminal amine moiety of G2 in conjunction with a metal ion and D233 activates the attacking water to generate the hydroxide ion nucleophile (21). Hence, the dynamics resulting in the disappearance of the N-terminal residues upon the addition of Ca^2+^ may be associated with this role. We also observed large chemical shift changes in response to addition of Ca^2+^ to the hFEN1–DNA complex in regions of the protein distal to the active site that were not observed at the same magnitude upon addition of Ca^2+^ to hFEN1. Several studies have shown that divalent metal ions and arch region ordering are necessary to move the scissile phosphate into the active site by untwisting (65) the reacting duplex (9,18,58,59). Thus, these large Ca^2+^ induced chemical shift changes in the H2tH motif, which directly contacts the reacting duplex 20 Å from the active site metal ions, are likely due the untwisting of the reacting duplex to move the scissile phosphate of into the active site. Remarkably, chemical shift perturbations are also propagated to the region around the 3′-flap binding pocket. The reappearance of S317 and other chemical shift changes in the 3′-flap binding region strongly suggests that the positioning of the 3′-flap duplex is altered upon the addition of Ca^2+^. Therefore, our study provides direct evidence of DNA-mediated allostery (66) between the active site and both reacting duplex and 3′-flap recognition, thereby linking regions involved in DNA structural recognition and catalysis.

When the 5′-flap is threaded through the helical arch, the top of α4 and α5 form a mini-hydrophobic core (21). The importance of this ordered state has previously been demonstrated; it is required for untwisting of the reacting duplex DNA to place the scissile phosphate on the active site while delivering two basic residues to the active site that are essential for biologically-relevant rates of reaction (11,18,59). Chemical shift analysis (53) of the NMR-observable region corresponding to the top of α4 (A107−A116) suggests that it is transiently sampling an α-helical conformation in its disordered state, whereas the corresponding region of α5 is not. This suggests that the disorder-to-order transition for α4 arises due to conformational selection (i.e., population shift), whereas the transition in α5 results from induced fit (67–69). Having a completely induced fit model for the arch would allow for enhanced specificity but would involve large entropic penalties and slow ordering. Using a conformational ensemble shift for the larger portion of the arch (α4) reduces these penalties. A potential trigger for the ensemble population shift for the α4 region could be the catalytically-crucial contact to the +1 phosphate of the 5′-flap as well as the formation of the mini-hydrophobic core (21). On the other side of the arch, several factors are likely to induce the disorder to α-helical transition of α5. These include interaction with the α2–α3 loop when it forms an Ω-loop (15) in response to the 3′-flap and electrostatic interaction of K125, K128 and R129 with template DNA phosphate diesters (11,21). Interestingly, the distance these lysine residues are from the template phosphate diesters is shorter when the arch moves forward towards the saddle (21).

Because hFEN1 requires a disordered region that can fold in response to the appropriate ligand, the evolutionary conservation of disorder and order promoting residues to maintain the delicate balance is necessary (55,70). CIDER (56) analyses of the arch suggest that substrate-free hFEN1 has evolved to populate an extended rather than a collapsed conformation. The extended conformation is necessary to allow DNA to thread. Seemingly innocuous mutations or even interaction with small molecules may alter the conformation of the disordered state (extended coil vs. collapsed) or change the balance between disorder and order, thereby inhibiting catalysis (55). Thus, the arch region and α2–α3 loop, which are not conserved with other superfamily members, could be potential drug targets for hFEN1 (71).

Why hFEN1 has evolved to have an intrinsically disordered region and loop to achieve catalysis may seem counterintuitive initially, because enzymology has traditionally focused on structure-function paradigms (70,71). However, there is a rationale for hFEN1 evolution of intrinsically disordered regions. Firstly, because hFEN1 needs to thread a 5′-flap through the helical arch, doing so when the arch is disordered would be easier due a larger capture radius and would provide the ability to accommodate 5′-flaps with some secondary structure. Secondly, the combination of induced fit and conformational selection with subsequent ensemble shifts for the arch allows fast ordering with low energy barriers in response to the appropriate substrate features necessary for specificity. However, there is also fast unfolding and release of incorrect binding partners (72). Thirdly, it allows for low affinity for the catalytically-relevant ensemble, which arises when the enthalpic gains in ordering are balanced by the entropic cost (70,71). Hence, specificity can be achieved while allowing the enzyme to disassociate once catalysis has occurred (i.e. turnover). Together, this work illustrates how conformational dynamic regions respond to appropriate substrate features, resulting in a shift in the ensemble towards the catalytically viable state (Figure 8).

Although all 5′-nuclease superfamily members recognize DNA junctions of some kind, each protein has its own specific DNA substrate (3). However, the use of flexible regions spanning a range of dynamic timescales for substrate recognition and control of active site assembly is likely to be a common theme in these structure-sensing nucleases. As hEXO1 also uses the threading mechanism to enforce substrate free 5′-ends (19,20) and requires a helical arch for active site assembly, it too is likely to use changes in protein dynamics for substrate accommodation and catalysis. In contrast, the Holiday junction resolvase GEN1 lacks the arch feature. A much shorter peptide linker that is the equivalent in GEN1 is not observable in current structures (23,24), despite containing superfamily conserved active site residues. This is the predicted dimerization interface, as two GEN1 monomers come together to sense the four-way-junction. Similarly, the very large equivalent of the arch in XPG, which also contains active site residues, presumably assembles as a result of the binding of other nucleotide excision repair protein partners and DNA (25). Thus, somewhat surprisingly, protein reversible plasticity appears to be the answer to coupling recognition with reaction for a range of nucleic acid hydrolases.

## DATA AVAILABILITY

The backbone ^1^H, ^15^N and ^13^C chemical shifts of the free protein in EDTA and the hFEN1_K93A_–DNA complex in Ca^2+^ have been deposited in the BioMagResBank (http://www.bmrb.wisc.edu/) under the BMRB accession codes 27160 and 27404, respectively.

## Supplementary Material

Supplementary Data
