# Supplementary material for: Regional conformational flexibility couples substrate specificity and scissile phosphate diester selectivity in human flap endonuclease 1
Source: Nucleic Acids Res. 2018 Apr 30;46(11):5618–33. doi: 10.1093/nar/gky293 (PMC6009646; doi:10.1093/nar/gky293)
Supplement: Supplementary Data [file gky293_supplemental_figures.pdf]

SUPPLEMENTARY DATA

Regional Intrinsic Disorder Couples Substrate Specificity and Scissile Phosphate Diester Selectivity in Human Flap Endonuclease 1

Ian A. Bennet, L. David Finger, Nicola J. Baxter, Benjamin Ambrose, Andrea M. Hounslow, Mark J. Thompson, Jack C. Exell, Nur Nazihah B. Md. Shahari, Timothy D. Craggs, Jonathan P. Waltho, Jane A. Grasby

Table S1

Oligonucleotide* sequences used for site directed mutagenesis of pET28b-hFEN1

NAME	SEQUENCE
**C235A_F**	5'gctaggcagtgactacgctgagagtatccggggt3'
**C235A_R**	5'accccggatactctcagcgtagtcactgcctagc3'
**C311A_F**	5'aagagctgatcaagttcatggctggtgaaaagcagttctctg3'
**C311A_R**	5'cagagaactgcttttcaccagccatgaacttgatcagctctt3'
**E120C_F**	5'aatttttccacctcctggcaggccccagcagcctgag3'
**E120C_R**	5'ctcaggctgctggggcctgccaggaggtggaaaaatt3'
**S293C_F**	5'agctccacacactctgggtccagcacc3'
**S293C_R**	5'ggtgctggacccagagtgtgtggagct3'

*Oligonucleotides were purchased from ThermoFisher without purification.

Table S2 (related to Figure 3)

Model selection for all 179 analyzed residues

Models	Number of Residues selected for each model
m1 = $\{S^2\}$	21
m2 = $\{S^2, \tau_e\}$	11
m3 = $\{S^2, R_{ex}\}$	62
m4 = $\{S^2, \tau_e, R_{ex}\}$	24
m5 = $\{S^2, S^2_f, \tau_s\}$	18
m6 = $\{S^2, \tau_f, S^2_f, \tau_s\}$	2
m7 = $\{S^2, S^2_f, \tau_s, R_{ex}\}$	28
m8 = $\{S^2, \tau_f, S^2_f, \tau_s, R_{ex}\}$	13
**TOTAL**	**179**

Table S3

Efficiency of fluorophore-maleimide labelling of $hFEN1_Q$ determined using UV/Vis spectrophotometry

Wavelength λ	$A_{\lambda max}$	$A_{\lambda max\ Corrected}$	Concentration#	[Chromophore]/[Protein] Ratio
280 nm	2.16	1.52*	66.19	1.00
559 nm	6.23	6.23‡	47.96	0.72
646 nm	7.05	7.05	47.00	0.71

*Corrected for Cy3b and ATTO 647N absorbtion at 280 nm

‡Corrected for ATTO 647N absorbtion at 559 nm

#Extinction coefficients used (ε_{280} = 2.292×10^4 M^{-1}cm^{-1}, ε_{559} = 1.3×10^5 M^{-1}cm^{-1}, ε_{646} = 1.5×10^5 M^{-1}cm^{-1})

Table S4

Parameters from fitting the smFRET data to a two Gaussian fit model (related to Figure 7)

Gauss Curve	Mean E	Sigma	Amplitude
**FEN1 Alone low FRET**	0.6455	0.1989	0.5304
**FEN1 Alone high FRET**	0.8762	0.0924	0.4428
**+20nM DNA low FRET**	0.6450	0.1990	0.4732
**+20nM DNA high FRET**	0.8760	0.0920	0.5044

Figure S1 (Related to Figure 1)

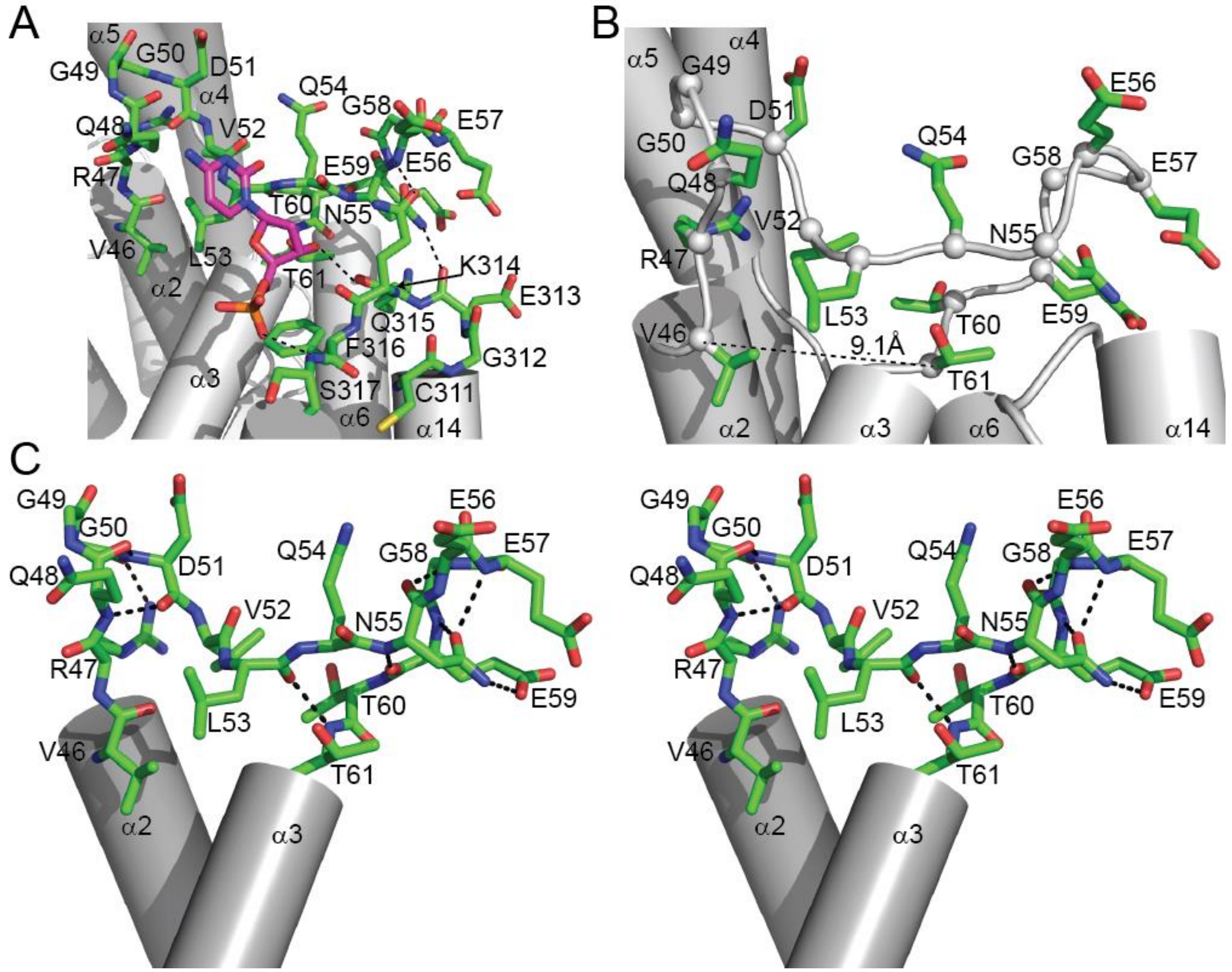

Figure S1. The 3'-flap is bound in a pocket made by the α14-α15 loop, the first turn of α15 and the α2-α3 loop, which conforms to the definition of an omega (Ω)-loop**.** (**A**) View of 3'-flap nucleotide of the DNA substrate (magenta) (3Q8K) cradled in its pocket (1). Residue identities and α-helices are indicated. Dashed lines indicate the hydrogen bonds formed between the α14–α15 and α2–α3 loops and the α14–α15 loop and the 3'-flap nucleotide. The N55 side chain amide forms a hydrogen bond with the E313 backbone carbonyl. The backbone amide of E56 forms a hydrogen bond with the side chain carbonyl of Q315. The backbone carbonyl of K314 forms a hydrogen bond with the 3'-hydroxyl moiety of the 3'-flap nucleotide. The backbone amide of S317 forms a hydrogen bond with a non-bridging phosphoryl oxygen atom of the phosphate diester moiety of the 3'-flap nucleotide. (**B**) Similar view as in panel A with the 3'-flap nucleotide and α14–α15 loop omitted for clarity. The traditional definition of an Ω-loop is a loop of 6 to 16 amino acid residues with the C^{α}–C^{α} distance of the loop termini both less than 10 Å (dashed line V46–T61 = 9.1 Å) and less than two-thirds the longest pairwise C^{α}–C^{α} distance in the loop (V46–E57 and G50–E57 = 18.4 Å). (**C**) Wall-eyed stereo-view of the α2-α3 loop to show the intra-loop network of hydrogen bonds among backbone and sidechain atoms when structured as an Ω-loop (2).

Figure S2 (Related to Figure 1)

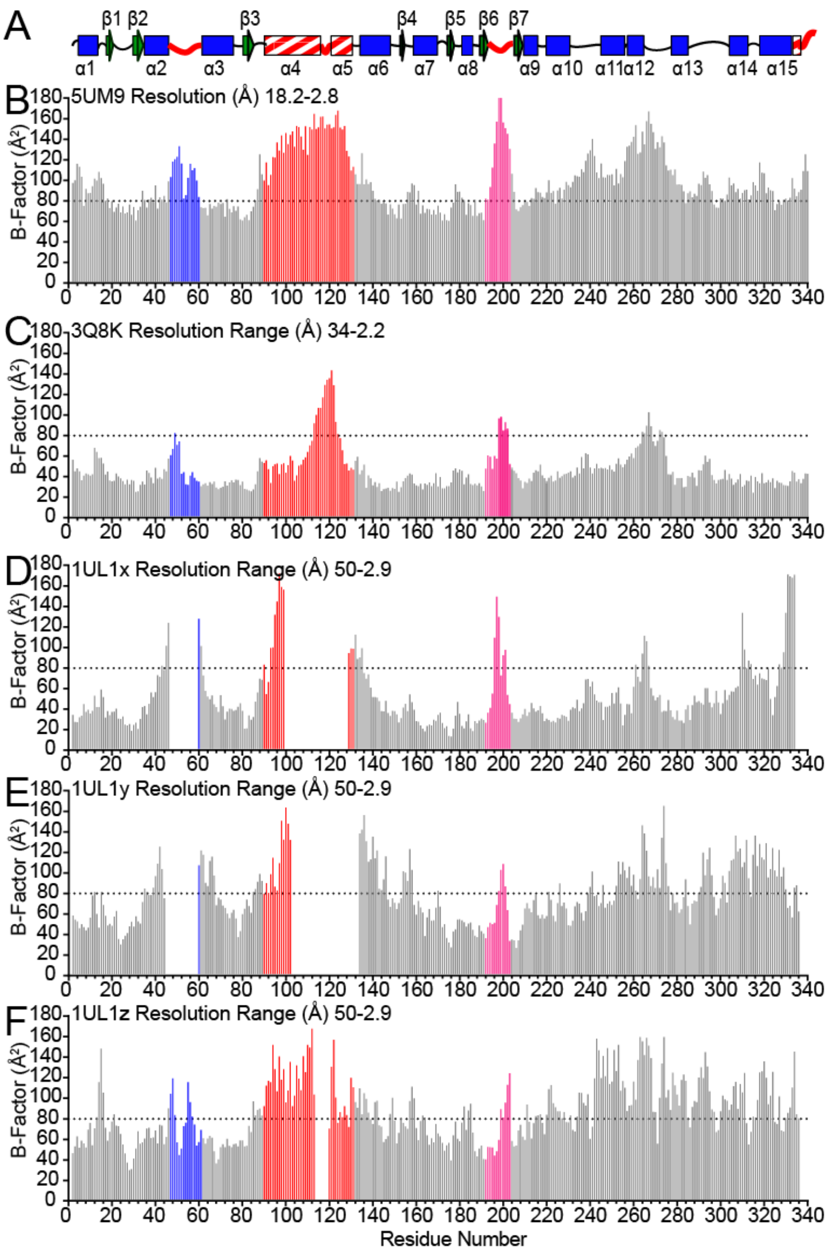

Figure S2. The α2-α3 loop, arch region, β-pin and the C-terminal residues consistently show higher than average temperature-factors (or B-factors). (**A**) Secondary structure schematic of hFEN1 from 3Q8K. Blue rectangles, green arrows and black lines indicate α-helices, β-strands, and loops, respectively. Loops known to have structural heterogeneity (1UL1 vs. 3Q8K) are indicated by red lines. Red and white stripped rectangles highlight regions where either α-helix or disorder has been observed. Plots of backbone amide nitrogen B-factors versus residue number for (**B**) 5UM9 (3), (**C**) 3Q8K (1), (**D**) 1UL1x, (**E**) 1UL1y and (**F**) 1UL1z (4). The α2-α3 loop, arch region, and β-pin are highlighted in blue, red and magenta, respectively. The dashed line indicates a B-factor of 80 Å^2, which is equivalent to a root mean square displacement of 1 Å.

Figure S3 (Related to Figure 1)

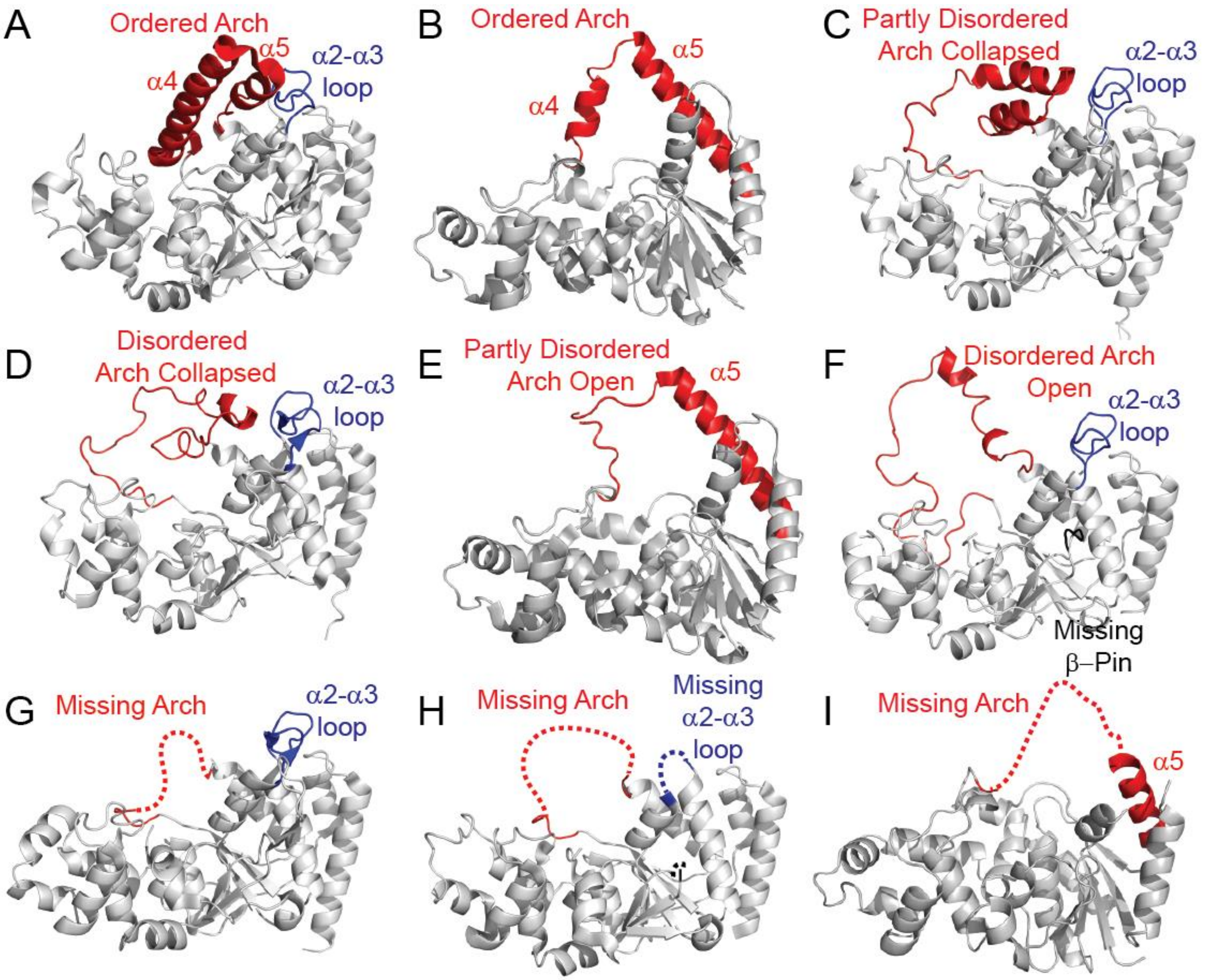

Figure S3. The arch regions and α2-α3 loops of FEN1 proteins from other organisms also show structural heterogeneity. Crystal structures of (**A**) *Desulfurococcus solfataricus* (3ORY) (7) and (**B**) several T5 FEN (1EXN, 1UT5 and 1UT8) (5,6) show an ordered arch region and α2-α3 loop. Two crystal structures of FEN1 from (**C**) *Pyrococcus furiosus* (1B43) (8) and (**D**) *Pyrococcus horikoshii* (1MC8) (9) show an ordered α2–α3 loop and an arch region with various degrees of secondary structure and random coil present, but it is collapsed over the active site. Crystal structures from (**E**) T5 FEN (5HML and 5HMM) (10) and (**F**) *Methanococcus jannaschii* (1A76 and 1A77) (11) show an ordered α2–α3 loop and an arch region with partly disordered areas, some visible secondary structure and an extended, open conformation. (**G**) The crystal structure of *Methanopyrus kandleri* (4WA8) (12) shows an ordered α2–α3 loop, but the arch region is completely missing suggesting disorder in these segments. The crystal structures of hFEN1 in complex with (**H**) an inhibitor (5FV7) (13) and (**i**) T5 FEN (1XO1) (14) lack density for both the arch region and the α2–α3 loop.

Figure S4 (Related to Figure 2)

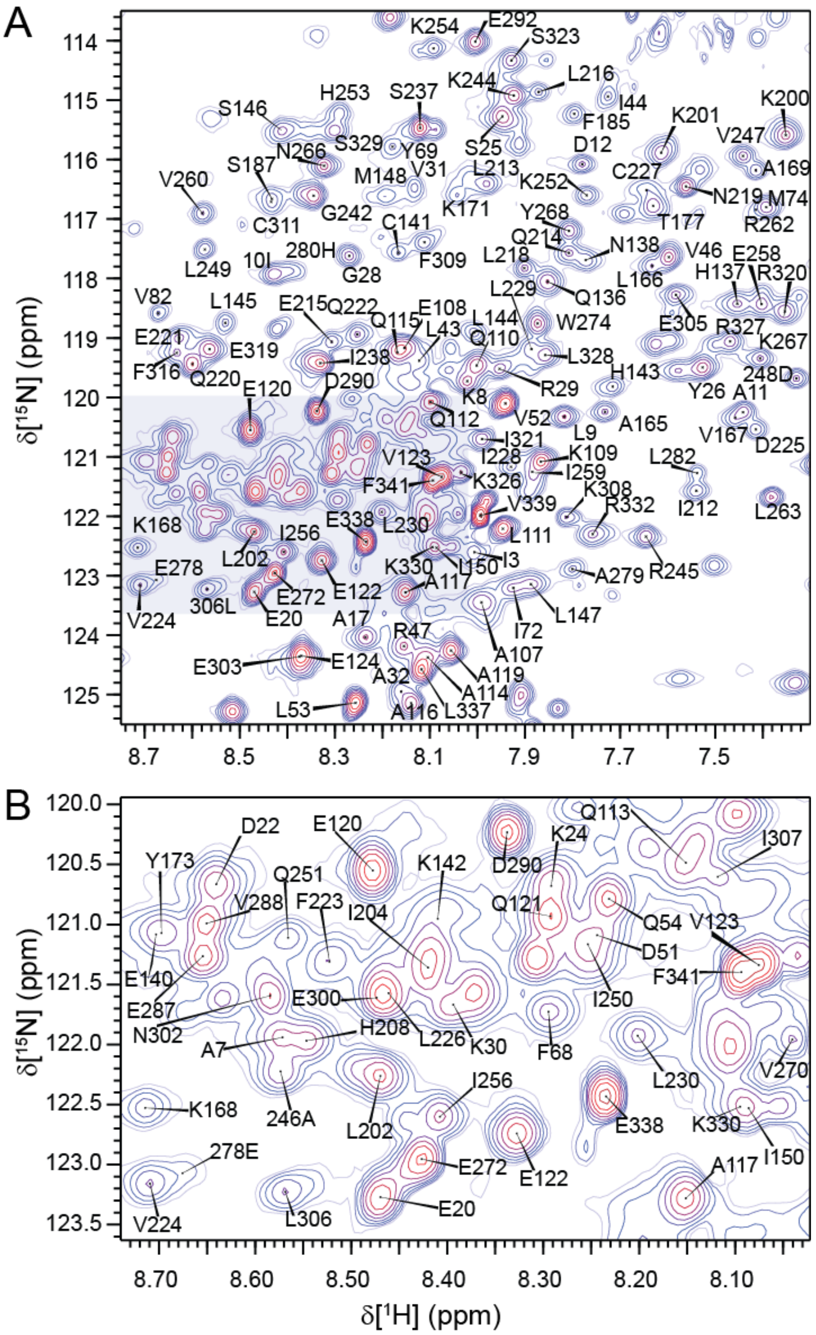

Figure S4. Expanded views of the crowded regions of the hFEN1 ^{1}H-^{15}N TROSY spectrum. (**A**) Expanded view of the shaded area in Figure 2A with assignments shown. (**B**) An expanded view of the shaded area in panel A with resonance assignments shown.

Figure S5 (Related to Figures1 and 2)

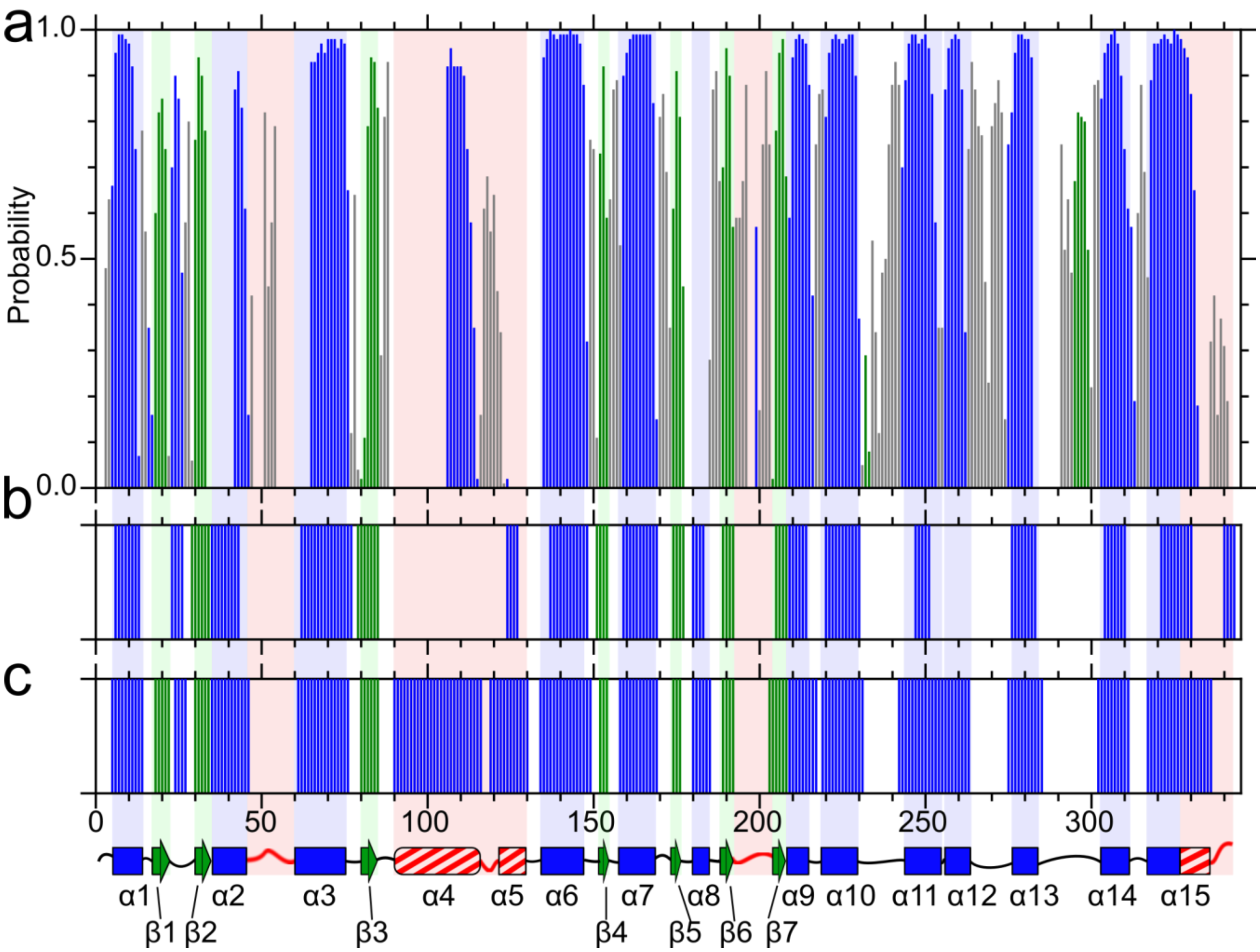

Figure S5. Secondary structure prediction for hFEN1 using backbone chemical shifts corresponds with the X-ray crystal structure of hFEN1 for the saddle region and the α2-α3 loop, but reveals differences in the arch region. (**A**) TALOS-N (15) secondary structure prediction based on assigned backbone chemical shifts ($^{1}H^{N}$, ^{15}N, $^{13}C^{\alpha}$, $^{13}C^{\beta}$, $^{13}C'$) for hFEN1. Predicted α-helices, β-strands and loops are shown as blue, green and gray bars, respectively. Absence of a bar in panel (**a**) indicates that the residue was not assigned. Secondary structure assignments for (**B**) 1UL1z (4) and (**c**) 3Q8K (1). Well-characterized α-helices and β-strands are shown, but loops are indicated by the absence of a bar in the panels (**B,C**). Secondary structure schematic derived from 3Q8K as described Figure S2A is included at the bottom of the figure.

Figure S6 (Related to Figure 3)

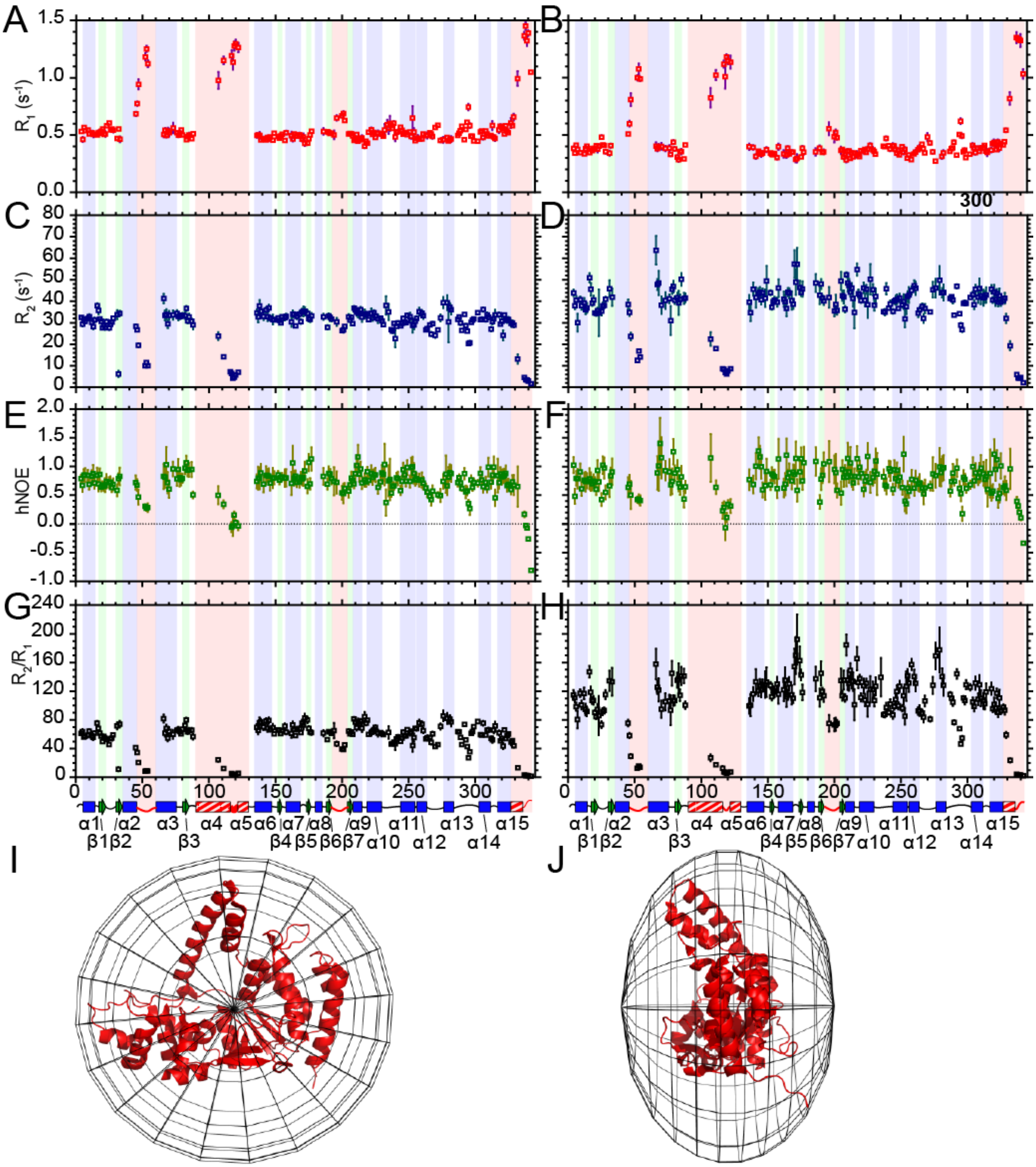

Figure S6. Experimentally-determined backbone ^{15}N relaxation parameters for hFEN1. (**A,B**) ^{15}N spin-lattice (R_1) rates, (**C,D**) spin-spin (R_2) rates and (**E,F**) ^{15}N-{^{1}H} NOE (hNOE) values at (**A,C,E**) 600 MHz and (**B,D,F**) 800 MHz for ^{2}H,^{15}N-labelled hFEN1 measured using interleaved TROSY-readout pulse sequences (16). R_2/R_1 plots generated from the data above for (**G**) 600 MHz and (**H**) 800 MHz, showing the per-residue average molecular correlation time in model-free methodology. Secondary structure maps are provided as described in Figure S2A. The oblate spheroid diffusion tensor best describes the tumbling of hFEN1 in solution. View of the diffusion tensor with the hFEN1 protein structure (3Q8K) (1) along the (**I**) z-axis and (**J**) x,y plane to illustrate agreement between the structure and the selected diffusion tensor.

Figure S7 (Related to Figure 3)

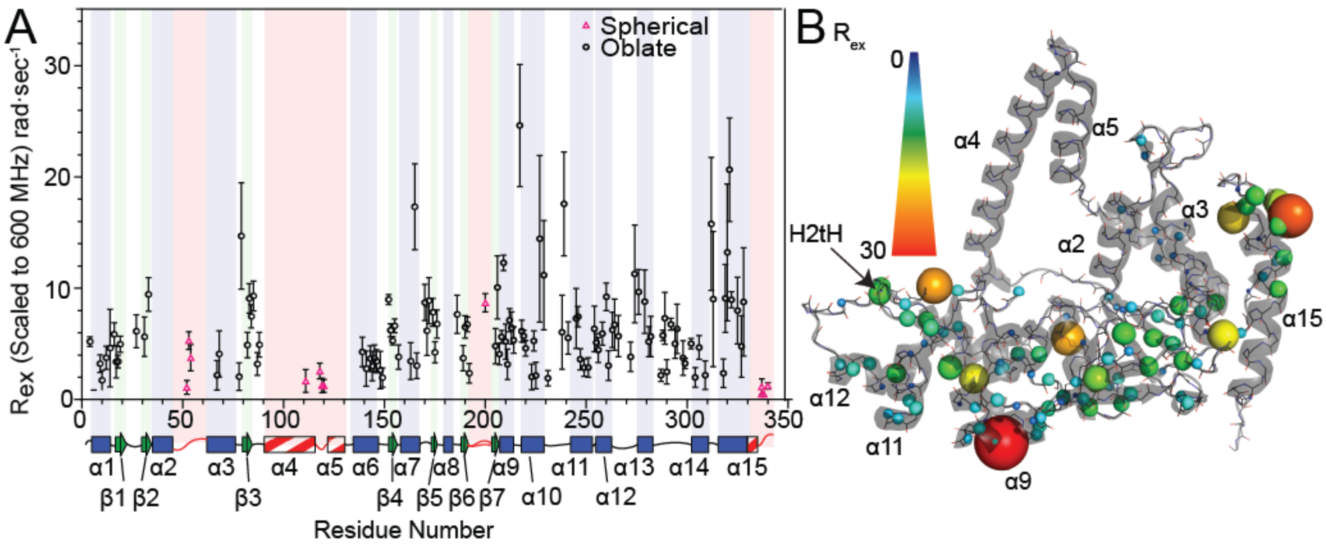

Figure S7. Model-free analysis of hFEN1 relaxation data identifies regions with millisecond timescale motions. (**A**) Chemical exchange terms (R_{ex}) were derived from *relax* (17) using backbone ^{15}N relaxation data acquired at 600 and 800 MHz (Figure S6A-F) and plotted versus residue number. Black circles represent data fitted to the oblate spheroid diffusion tensor (Figure 6I and J), whereas pink triangles were fitted to a spherical diffusion tensor. Locations of the residues with respect to protein secondary structure are illustrated below the panel as described in Figure S2A. (**B**) R_{ex} values plotted on a cartoon depiction of the hFEN1 protein structure (3Q8K) (1). The spheres represent the nitrogen nuclei for which data were derived. The R_{ex} spectrum bars illustrate the magnitude of R_{ex} values with respect to sphere color and size.

Figure S8 (Related to Figures 5 and 6)

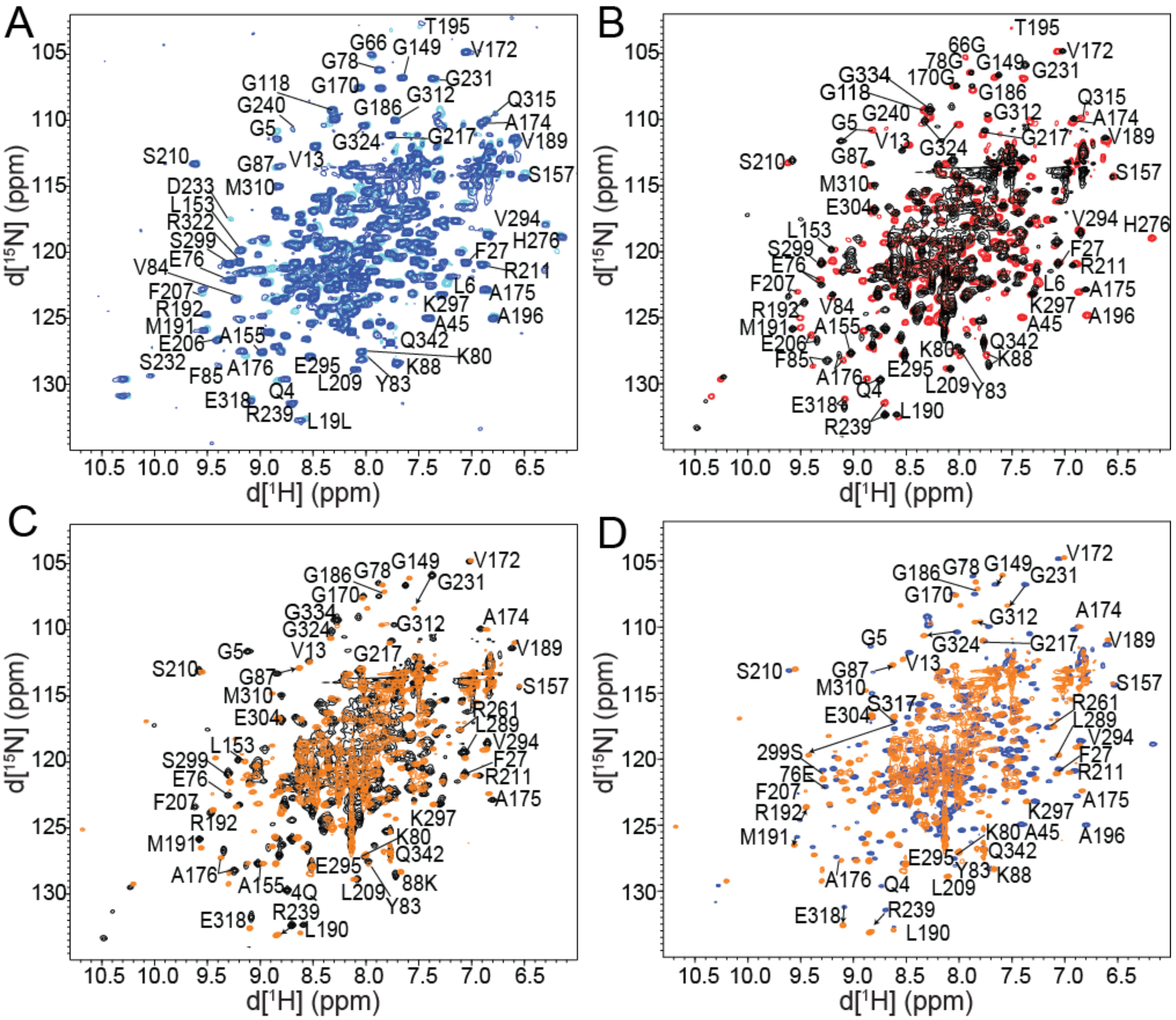

Figure S8. Superposed ^{1}H-^{15}N TROSY spectra illustrating chemical shift changes that arise from the addition of divalent cations to hFEN1, hFEN1$_{K93A}$ and the hFEN1$_{K93A}$-DNA complex. (**A**) ^{1}H-^{15}N TROSY spectra of hFEN1 in the presence of 8 mM Mg^{2+} (cyan) and 8 mM Ca^{2+} (blue) are nearly identical. Minor differences are seen for residues close to the active site (e.g., Q4, G5, L6, S157 and A176) and may arise due to the larger size and looser coordination geometry of Ca^{2+}. These small changes indicate that the Mg^{2+} and Ca^{2+} divalent metal ions are coordinated in the same active site locations. (**B**) ^{1}H-^{15}N TROSY spectra of hFEN1$_{K93A}$ (red) and the hFEN1$_{K93A}$-DNA complex (black) in the presence of 0.1 mM EDTA. (**C**) ^{1}H-^{15}N TROSY spectra of the hFEN1$_{K93A}$-DNA complex in the presence of 0.1 mM EDTA (black) or 8 mM Ca^{2+} (orange). (**D**) ^{1}H-^{15}N TROSY spectra of hFEN1$_{K93A}$ (blue) and the hFEN1$_{K93A}$-DNA complex (orange) in the presence of 8 mM Ca^{2+}. Peaks with good chemical shift dispersion are labelled.

Figure S9 (Related to Figures 6 and 7)

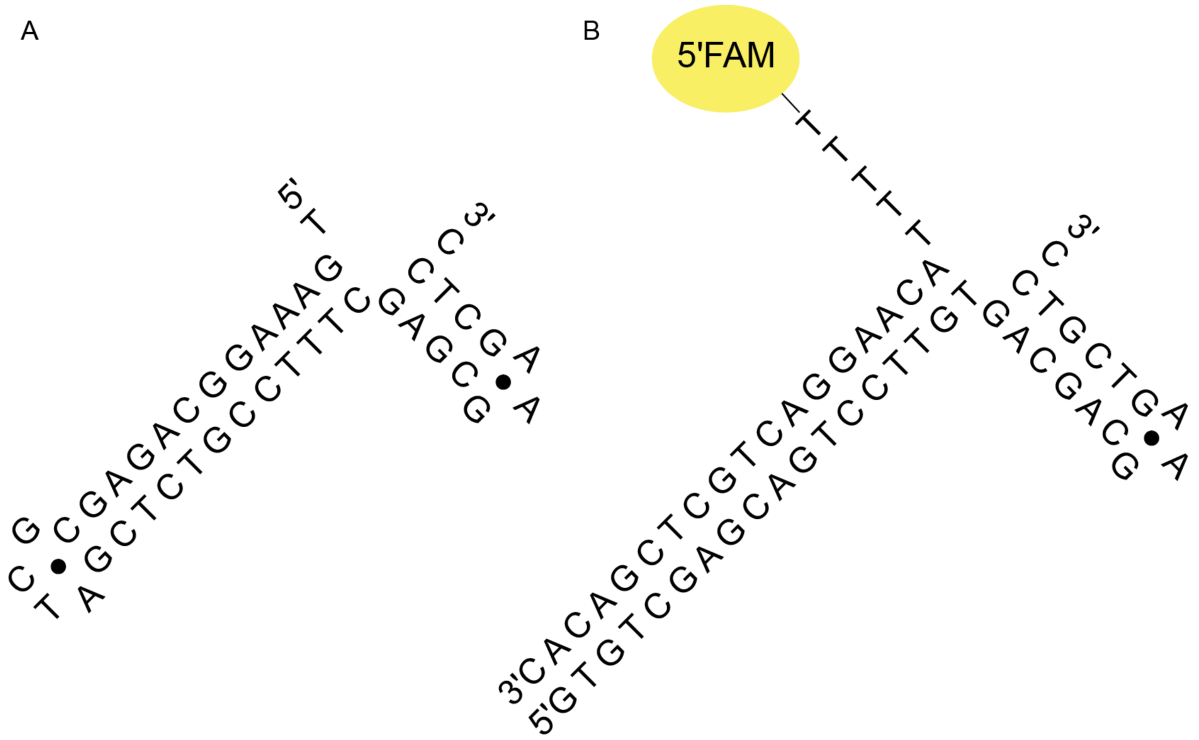

Figure S9. Sequence and secondary structure of the DNA substrate used herein. (**A**) The hFEN1$_{K93A}$-DNA complex uses a unimolecular substrate design bearing stable tri- (dGAA) (18) and tetra- (dGCTA) (19) loops to prevent hFEN1 inadvertently binding blunt-ended duplex. (**B**) The bimolecular substrate was used to assess the initial rates of reaction used to determine second order rate constants. In addition, this same substrate was used in smFRET experiments except it lacked the 5'-FAM label.

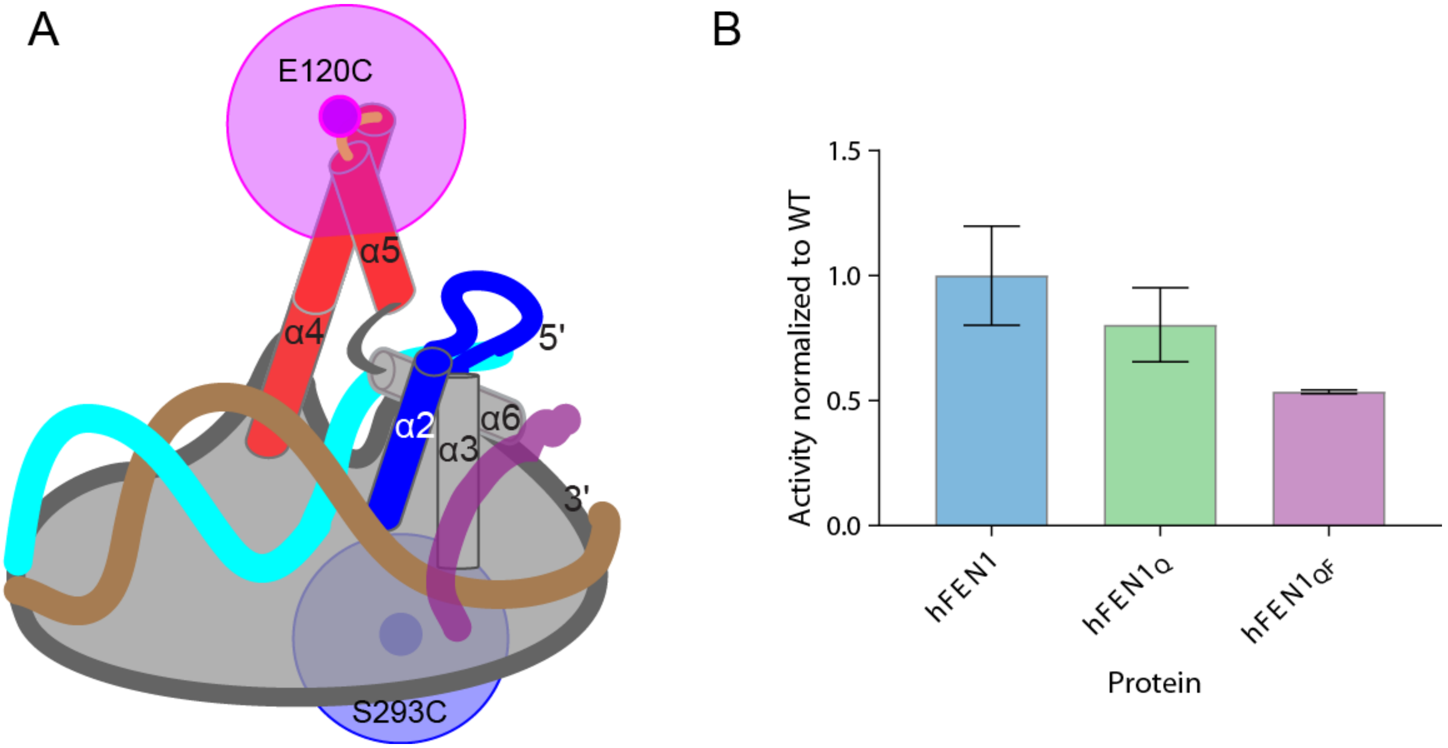

Figure S10. Relative labelling positions, activity and mass spectrometry results for hFEN1$_{Q}$ and hFEN1$_{QF}$ proteins. (**A**) Relative labelling positions of the fluorophores on hFEN1$_{QF}$ with substrate. (**B**) Activity of hFEN1 proteins assessed by normalized initial rate measurements at 50 nM substrate relative to WT protein. Values represent the average of three measurements (n=3), and error bars represent the standard error of the mean (SEM).

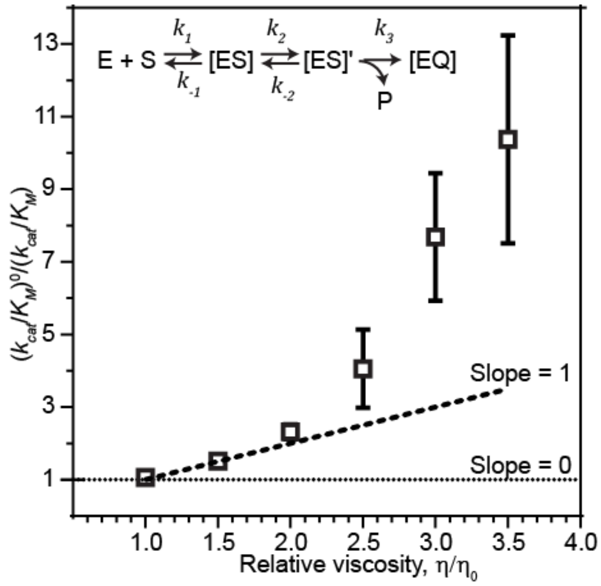

Figure S11. The effect of viscogen on relative second order rate constants is larger than expected and non-linear. Graph of mean relative second order rate constants $(k_{cat}/K_M)_0/(k_{cat}/K_M)$ for hFEN1-catalyzed hydrolysis of DF5,1 (Figure S9B) versus relative viscosity (SEM, n=3) shows a non-linear dependence. Inset: simplified scheme of the hFEN1-catalyzed reaction illustrating bimolecular complex [ES] formation, a conformational change to the catalytically-competent state [ES]' and substrate hydrolysis into ssDNA (P) and enzyme-dsDNA [EQ] products. Microrate constants (*k*) up to and including the first irreversible step (k_3) contribute to k_{cat}/K_M. hFEN1 second-order reaction rates (k_{cat}/K_M) on double-flap DNA substrates approached diffusion-control (10^7–10^9 $M^{-1}\cdot s^{-1}$); therefore, hFEN1 has likely achieved catalytic perfection with its optimal substrate (Figure S9B) (20). Observation of a linear dependence for the reciprocal of relative k_{cat}/K_M with respect the relative viscosity of the solution has been the standard manner by which to confirm catalytic perfection or diffusion-control for enzymes (21,22). Assuming that only the rates of enzyme and substrate association and complex disassociation are affected by viscogen, a linear relationship is expected for the reciprocal of normalized k_{cat}/K_m with respect to relative viscosity. Moreover, a diffusion-controlled reaction is expected to afford a slope of one (dashed line), whereas an enzyme that is not diffusion controlled is expected to afford a line with a slope of zero (dotted line) (21,22). Although increasing the relative viscosity of the reaction buffer with either glycerol or sucrose did increase the reciprocal of normalized k_{cat}/K_m with the optimal double flap substrate, the effects were non-linear especially at relative viscosities greater than two. Similar non-linear viscogen dependence has been reported for an intrinsically disordered protein that folds upon binding its protein partner (23). In addition to slowing diffusional encounter, microviscogens have also been shown to slow protein conformational rearrangements (24) and protein-catalyzed reactions when conformational changes in the catalytic cycle were involved (25). Indeed, the impact of viscogen on rates of hFEN1-catalyzed reaction under single-turnover conditions measured by single molecule techniques is consistent with viscogen slowing conformational change steps after enzyme-substrate complex formation (26). Therefore, the non-linear viscogen dependence on relative second order rate constants could be due to the combined effect of viscogen on the rates of bimolecular association and subsequent conformational changes. However, we cannot rule out the possibility that sucrose and glycerol are acting either as inhibitors of the reaction by some unknown mechanism or that these polyols are affecting the energy landscape of the various conformational ensembles by acting as osmolytes.

SUPPLEMENTARY REFERENCES

...1. Tsutakawa, S.E., Classen, S., Chapados, B.R., Arvai, A.S., Finger, L.D., Guenther, G., Tomlinson, C.G., Thompson, P., Sarker, A.H., Shen, B.i. *et al.* (2011) Human flap endonuclease structures, DNA double-base flipping, and a unified understanding of the FEN1 superfamily. *Cell*, **145**, 198-211.

...2. Fetrow, J.S. (1995) Omega loops: nonregular secondary structures significant in protein function and stability. *FASEB J.*, **9**, 708-717.

...3. Tsutakawa, S.E., Thompson, M.J., Arvai, A.S., Neil, A.J., Shaw, S.J., Algasaier, S.I., Kim, J.C., Finger, L.D., Jardine, E., Gotham, V.J.B. *et al.* (2017) Phosphate steering by Flap Endonuclease 1 promotes 5′-flap specificity and incision to prevent genome instability. *Nat. Commun.*, **8**, 15855.

...4. Sakurai, S., Kitano, K., Yamaguchi, H., Hamada, K., Okada, K., Fukuda, K., Uchida, M., Ohtsuka, E., Morioka, H. and Hakoshima, T. (2005) Structural basis for recruitment of human flap endonuclease 1 to PCNA. *EMBO J.*, **24**, 683-693.

...5. Ceska, T.A., Sayers, J.R., Stier, G. and Suck, D. (1996) A helical arch allowing single-stranded DNA to thread through T5 5'-exonuclease. *Nature*, **382**, 90-93.

...6. Feng, M., Patel, D., Dervan, J.J., Ceska, T., Suck, D., Haq, I. and Sayers, J.R. (2004) Roles of divalent metal ions in flap endonuclease-substrate interactions. *Nat. Struct. Mol. Biol.*, **11**, 450-456.

...7. Mase, T., Kubota, K., Miyazono, K., Kawarabayasi, Y. and Tanokura, M. (2009) Crystallization and preliminary X-ray analysis of flap endonuclease 1 (FEN1) from Desulfurococcus amylolyticus. *Acta Crystallogr Sect F Struct Biol Cryst Commun*, **65**, 923-925.

...8. Hosfield, D.J., Mol, C.D., Shen, B. and Tainer, J.A. (1998) Structure of the DNA repair and replication endonuclease and exonuclease FEN-1: coupling DNA and PCNA binding to FEN-1 activity. *Cell*, **95**, 135-146.

...9. Matsui, E., Musti, K.V., Abe, J., Yamasaki, K., Matsui, I. and Harata, K. (2002) Molecular structure and novel DNA binding sites located in loops of flap endonuclease-1 from Pyrococcus horikoshii. *J Biol Chem*, **277**, 37840-37847.

...10. AlMalki, F.A., Flemming, C.S., Zhang, J., Feng, M., Sedelnikova, S.E., Ceska, T., Rafferty, J.B., Sayers, J.R. and Artymiuk, P.J. (2016) Direct observation of DNA threading in flap endonuclease complexes. *Nat. Struct. Mol. Biol.*, **23**, 640-646.

...11. Hwang, K.Y., Baek, K., Kim, H.-Y. and Cho, Y. (1998) The crystal structure of flap endonuclease-1 from Methanococcus jannaschii. *Nat. Struct. Mol. Biol.*, **5**, 707-713.

...12. Shah, S., Dunten, P., Stiteler, A., Park, C.K. and Horton, N.C. (2015) Structure and specificity of FEN-1 from Methanopyrus kandleri. *Proteins*, **83**, 188-194.

...13. Exell, J.C., Thompson, M.J., Finger, L.D., Shaw, S.J., Debreczeni, J., Ward, T.A., McWhirter, C., Sioberg, C.L., Martinez Molina, D., Abbott, W.M. *et al.* (2016) Cellularly active N-hydroxyurea FEN1 inhibitors block substrate entry to the active site. *Nat. Chem. Biol.*, **12**, 815-821.

...14. Garforth, S.J., Ceska, T.A., Suck, D. and Sayers, J.R. (1999) Mutagenesis of conserved lysine residues in bacteriophage T5 5′-3′ exonuclease suggests separate mechanisms of endoand exonucleolytic cleavage *Proc. Natl. Acad. Sci. USA*, **96**, 38-43.

...15. Shen, Y. and Bax, A. (2013) Protein backbone and sidechain torsion angles predicted from NMR chemical shifts using artificial neural networks. *J. Biomol. NMR*, **56**, 227-241.

...16. Lakomek, N.A., Ying, J. and Bax, A. (2012) Measurement of ^{15}N relaxation rates in perdeuterated proteins by TROSY-based methods. *J. Biomol. NMR*, **53**, 209-221.

...17. Bieri, M., d'Auvergne, E.J. and Gooley, P.R. (2011) relaxGUI: a new software for fast and simple NMR relaxation data analysis and calculation of ps-ns and mus motion of proteins. *J. Biomol. NMR*, **50**, 147-155.

...18. Yoshizawa, S., Kawai, G., Watanabe, K., Miura, K.-i. and Hirao, I. (1997) GNA Trinucleotide Loop Sequences Producing Extraordinarily Stable DNA Minihairpins. *Biochemistry*, **36**, 4761-4767.

...19. Nakano, M., Moody, E.M., Liang, J. and Bevilacqua, P.C. (2002) Selection for thermodynamically stable DNA tetraloops using temperature gradient gel electrophoresis reveals four motifs: d(cGNNAg), d(cGNABg),d(cCNNGg), and d(gCNNGc). *Biochemistry*, **41**, 14281-14292.

...20. Finger, L.D., Blanchard, M.S., Theimer, C.A., Sengerová, B., Singh, P., Chavez, V., Liu, F., Grasby, J.A. and Shen, B. (2009) The 3′-flap pocket of human flap endonuclease 1 is critical for substrate binding and catalysis. *J. Biol. Chem.*, **284**, 22184-22194.

...21. Brouwer, A.C. and Kirsch, J.F. (1982) Investigation of diffusion-limited rates of chymotrypsin reactions by viscosity variation. *Biochemistry*, **21**, 1302-1307.

...22. Sengerova, B., Tomlinson, C., Atack, J.M., Williams, R., Sayers, J.R., Williams, N.H. and Grasby, J.A. (2010) Bronsted analysis and rate-limiting steps for the T5 flap endonuclease catalyzed hydrolysis of exonucleolytic substrates. *Biochemistry*, **49**, 8085-8093.
...23. Rogers, J.M., Steward, A. and Clarke, J. (2013) Folding and binding of an intrinsically disordered protein: fast, but not 'diffusion-limited'. *J. Am. Chem. Soc.*, **135**, 1415-1422.
...24. Sekhar, A., Latham, M.P., Vallurupalli, P. and Kay, L.E. (2014) Viscosity-dependent kinetics of protein conformational exchange: microviscosity effects and the need for a small viscogen. *J Phys Chem B*, **118**, 4546-4551.
...25. Rauscher, A., Derenyi, I., Graf, L. and Malnasi-Csizmadia, A. (2013) Internal friction in enzyme reactions. *IUBMB Life*, **65**, 35-42.
...26. Rashid, F., Harris, P.D., Zaher, M.S., Sobhy, M.A., Joudeh, L.I., Yan, C., Piwonski, H., Tsutakawa, S.E., Ivanov, I., Tainer, J.A. *et al.* (2017) Single-molecule FRET unveils induced-fit mechanism for substrate selectivity in flap endonuclease 1. *eLife*, **6**, e21884.
